# Maleidride biosynthesis – construction of dimeric anhydrides – more than just heads or tails

**DOI:** 10.1039/d2np00041e

**Published:** 2022-09-21

**Authors:** Katherine Williams, Agnieszka J. Szwalbe, Kate M. J. de Mattos-Shipley, Andy M. Bailey, Russell J. Cox, Christine L. Willis

**Affiliations:** a School of Biological Sciences, Life Sciences Building, University of Bristol 24 Tyndall Ave Bristol BS8 1TQ UK katherine.williams@bristol.ac.uk; b Celon Pharma Warsaw Poland; c Institute for Organic Chemistry and BMWZ, Leibniz University of Hannover Schneiderberg 38 30167 Hannover Germany; d School of Chemistry, University of Bristol Cantock's Close Bristol BS8 1TS UK

## Abstract

Covering: up to early 2022

Maleidrides are a family of polyketide-based dimeric natural products isolated from fungi. Many maleidrides possess significant bioactivities, making them attractive pharmaceutical or agrochemical lead compounds. Their unusual biosynthetic pathways have fascinated scientists for decades, with recent advances in our bioinformatic and enzymatic understanding providing further insights into their construction. However, many intriguing questions remain, including exactly how the enzymatic dimerisation, which creates the diverse core structure of the maleidrides, is controlled. This review will explore the literature from the initial isolation of maleidride compounds in the 1930s, through the first full structural elucidation in the 1960s, to the most recent *in vivo*, *in vitro*, and *in silico* analyses.

## Abstract 2

We review studies from early isolation and elucidation to recent bioinformatics and *in vitro* work regarding the biosynthesis of maleidrides. These are dimeric anhydrides, made from monomers coupled in various modes leading to structural diversity.

## Graphical abstract

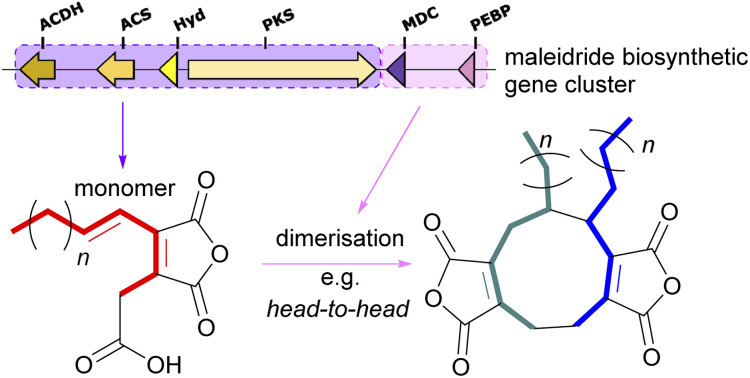

## 1. Introduction

**Fig. 1:**
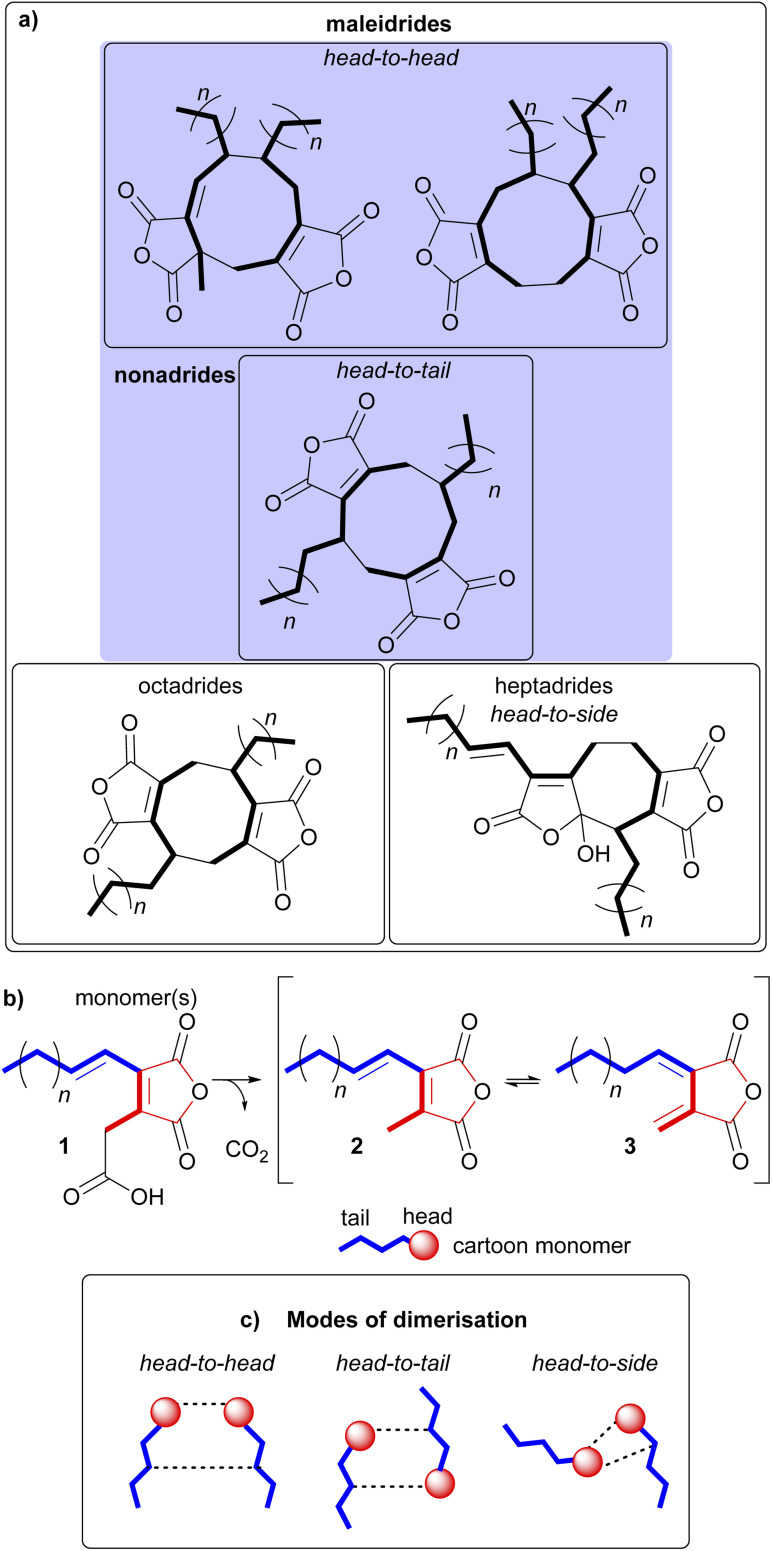
(a) Examples of the core dimeric structures of the maleidrides. (b) The three maleidride monomers, with the ‘tail’ depicted in blue, and the ‘head’ in red. (c) A pictorial representation of the various modes of dimerisation.

Maleidrides are a group of biosynthetically related polyketide-based natural products that have been isolated from diverse filamentous fungi.^1,2^ They contain at least one maleic anhydride moiety fused to a central carbocyclic core. There are three groups of maleidrides classified by the number of carbons in the central ring structure, the nonadrides (nine carbons), octadrides (eight carbons) and heptadrides (seven carbons) (Fig. 1A).^1^ Other maleic anhydride based metabolites are known,^2^ for example the cordyandhydrides^3^ and the tropolones.^4^ However, maleidrides are specifically formed by the coupling of two monomer units (1–3, Fig. 1B) to form a central carbocycle, with differing regiochemical dimerisation modes leading to significant structural diversity (Fig. 1C).^5–7^ Dimerisation is proposed to occur in a head-to-head, head-to-tail, or head-to-side manner leading to the observed maleidride core structures (Fig. 1). The initial position of the pendant alkyl chains varies dependent on the mode of dimerisation, with head-to-head coupling leading to neighbouring side chains and head-to-tail to side chains on opposite sides of the central carbocycle (see Fig. 1A, Sections 5.1 and 5.2 for further details). Further tailoring modifications and rearrangements increase the structural complexity of maleidride natural products and can influence their bioactive properties.

The numbering systems used for the maleidrides varies greatly in the literature and shows no consistency. Hence in 2020 we proposed a more systematic method based on the size of the ring (1–9, 1–8, 1–7 as appropriate) beginning at the carbon alpha to the maleic anhydride ring, which gives the lowest numbers to the side chains. The maleic anhydride carbons are numbered with a prime, appropriate to the ring numbering, hence 3′ 4′ and 8′ 9′ for byssochlamic acid, and 1′′, 2′′, *etc.* for the first side chain, numbering from the ring junction, and 1′′′, 2′′′, *etc.* for the second chain.^8^ We have used this numbering system throughout.

This review aims to bring together studies on the chemical, genetic, and enzymatic aspects of maleidride biosynthesis. We will explore the literature regarding the biosynthesis of the monomer, evidence for dimerisation, and maleidride tailoring, by reviewing feeding studies, biomimetic syntheses, bioinformatics, gene deletions, heterologous expression and *in vitro* enzyme assays.

## 2. Maleidride structures and their bioactivities

### 2.1. Nonadrides

**Fig. 2:**
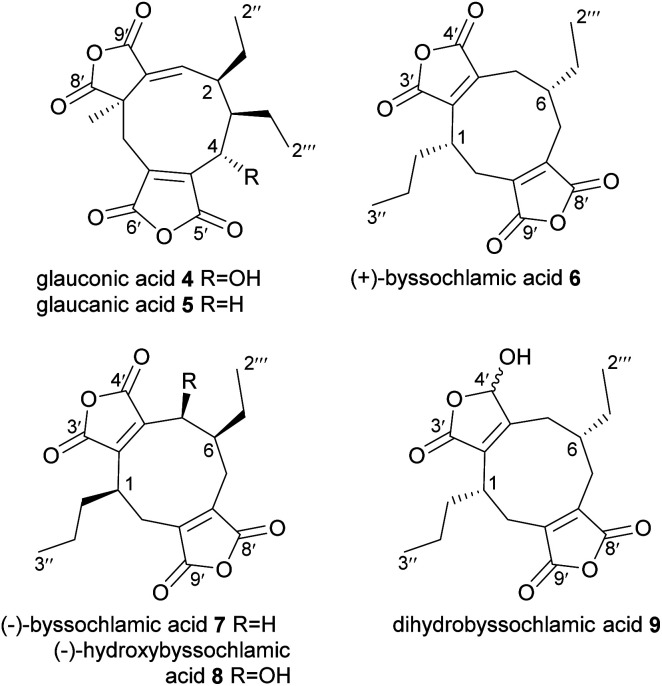
Nonadrides 4–9, with carbons numbered according to the system described in de Mattos-Shipley *et al.*^8^

In 1931 Wijkman and co-workers isolated the first maleidrides from culture extracts of *Penicillium glaucum*, glauconic and glaucanic acids 4 and 5, (Fig. 2).^9^ Soon after, an isomer of glaucanic acid 5, (+)-byssochlamic acid 6 was isolated from *Paecilomyces fulvus*, a common contaminant of pasteurised goods.^10^ In the 1960s full structural elucidation of these compounds was achieved through both chemical degradation studies and X-ray crystallography.^11–15^ In 1965, Barton and Sutherland named this family of related compounds (4–6) the ‘nonadrides’ in reference to the C_9_-monomers thought to be involved in their construction,^5^ however this name has later become associated with the number of carbons in the central carbocyclic core of the maleidrides.

None of the initially discovered nonadrides have shown any significant bioactivities.^16,17^ Many years later, (−)-byssochlamic acid 7, along with (−)-hydroxybyssochlamic acid 8 were extracted from a fungus that was isolated from a mangrove swamp.^18,19^ (−)-Byssochlamic acid 7 was shown to have medium cytotoxic activity against HEp-2 and HepG2 cells, whereas (−)-hydroxybyssochlamic acid 8 showed weak activity.^19^ A reduced derivative of (+)-byssochlamic acid, dihydrobyssochlamic acid 9 was isolated from *P. fulvus* in 2015 (Fig. 2).^1^

**Fig. 3:**
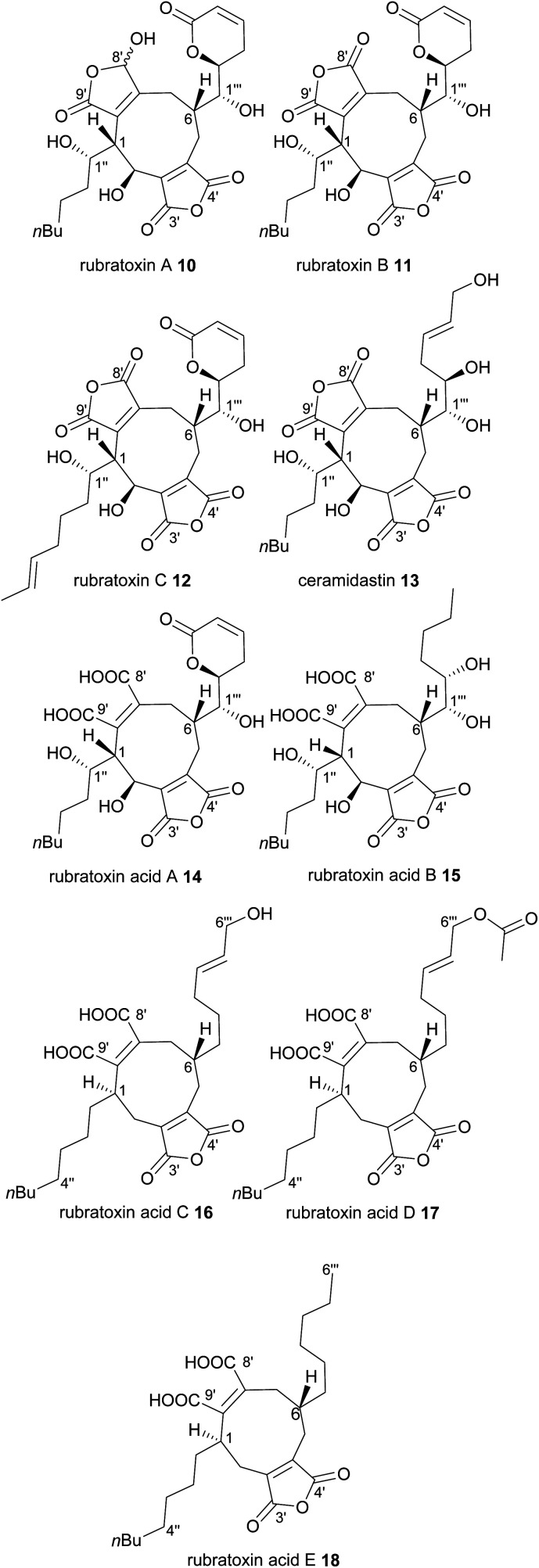
Nonadrides 10–18, with carbons numbered according to the system described in de Mattos-Shipley *et al.*^8^

The rubratoxins A and B, 10 and 11 were first isolated from *Penicillium rubrum* in 1962,^20^ and identified as the likely causative agents of fatal hepatotoxic poisoning events that occurred from contaminated foodstuffs. By 1970 their structures had been elucidated using a combination of degradation studies and X-ray crystallography, with the only difference between A and B being the reduction of one maleic anhydride moiety to a γ-hydroxybutenolide in rubratoxin A 10 (Fig. 3).^21–24^ These compounds are strikingly more complex than the nonadrides (4–9) that had been previously characterised, and also the first nonadrides which appear to be formed not from two C_9_-monomers, but instead by coupling of C_13_-units. Despite their complexity, it is apparent that the mode of dimerisation is head-to-tail coupling, as occurs in byssochlamic acid 6 biosynthesis, as their pendant alkyl chains are positioned on opposite sides of the central carbocycle (Fig. 1 and 3). A desaturated derivative of rubratoxin B 11, rubratoxin C 12 was later isolated from a *Penicillium* sp.^25^ Rubratoxin A 10 is a potent and highly specific inhibitor of protein phosphatase 2A, (PP2A), a target for anticancer drug development. Notably, it has approximately 100-fold stronger inhibition of PP2A than rubratoxin B 11.^26^ The γ-hydroxybutenolide motif has been shown to be an important pharmacophore in other compounds.^27–29^ Rubratoxin B 11 exhibits antitumour activity, likely linked to blocks in the progression of the cell cycle.^30^ Rubratoxin C 12 shows weak activity against human cancer cell lines.^25^ Ceramidastin 13, an analogue of the rubratoxins, has been isolated, also from a *Penicillium* sp.^31^ Inoue *et al.*^31^ state that the ^1^H and ^13^C chemical shifts and coupling constants of ceramidastin 13 were very similar to those reported for rubratoxin B 11, suggesting the same stereochemistry between the two compounds, as shown in Fig. 3. Ceramidastin 13 was shown to be a novel inhibitor of bacterial ceramidase,^31^ an enzyme which is believed to contribute to skin infections of patients with atopic dermatitis.^32^ In 2019, a rubratoxin producing fungus, *Talaromyces purpurogenus*^33^ was shown to produce five other nonadride compounds (14–18), one of which is an analogue of rubratoxin B 11 with one of the maleic anhydride moieties hydrolysed to a diacid (rubratoxin acid A 14).^34^ Maleic anhydride ring-open forms of nonadrides may be artefacts of extraction protocols, and are known to interconvert with the ring-closed forms.^1,35,36^ Hence it is difficult to determine whether 14 is a true natural product, although the authors note that 14 appears stable in their hands.^34^ Compounds 15, 16, 17 and 18 also all contain one ring-open diacid and appear to be intermediates/shunts from the rubratoxin pathway.^37^ All five compounds (14–18) were tested for their *in vitro* anti-inflammatory activities, with rubratoxin acid A 14 showing significant inhibitory activity against nitric oxide production (thought to play a crucial role in inflammatory responses)^38^ from liposaccharide (LPS)-induced RAW264.7 cells.^34^

**Fig. 4:**
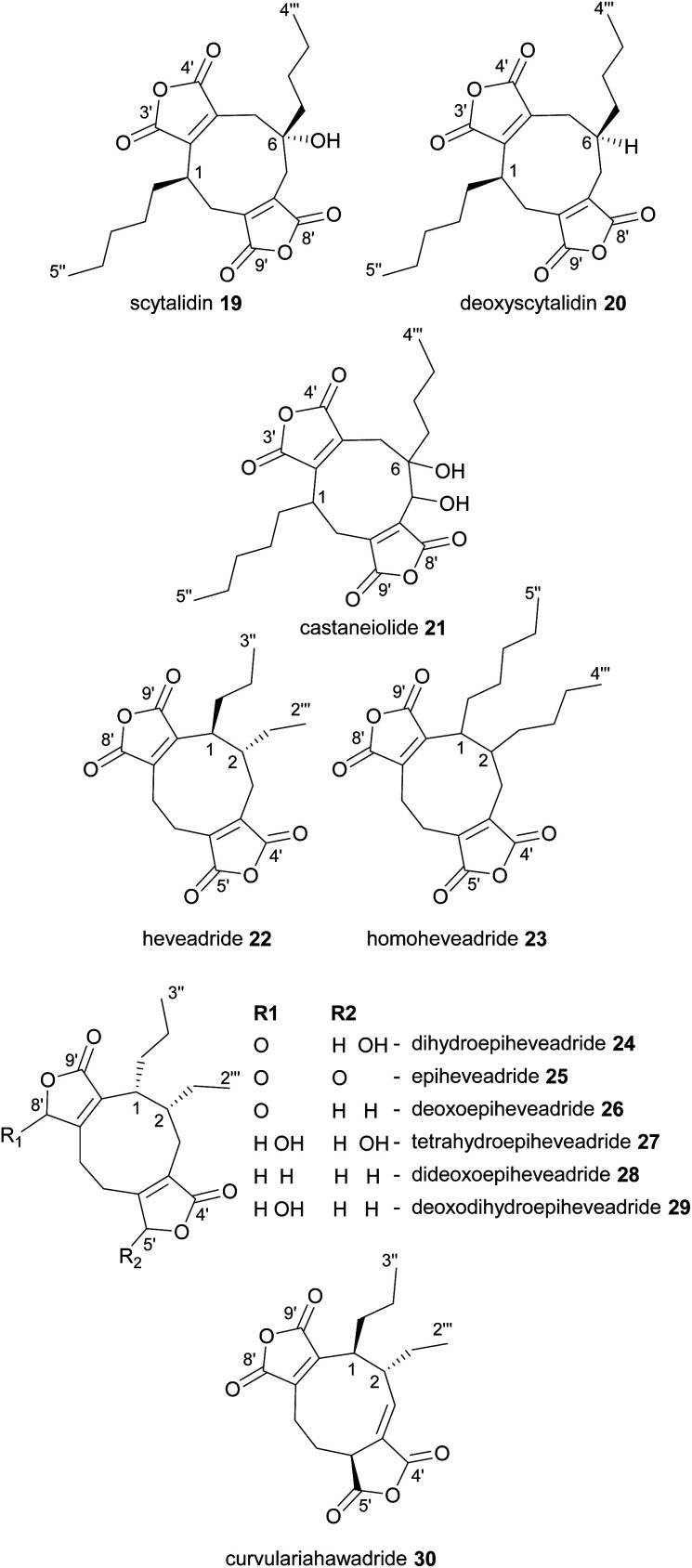
Nonadrides 19–30, with carbons numbered according to the system described in de Mattos-Shipley *et al.*^8^

In 1972 scytalidin 19 was isolated from a *Scytalidium* species and characterised, however the relative and absolute configurations were not determined.^39^ Later analysis of various *Scytalidium* species revealed that deoxyscytalidin 20 is also produced by scytalidin 19 producers.^40^ Nonadrides 19 and 20 possess the same ring structure as byssochlamic acid 6, but with longer alkyl chains, providing further confirmation that the maleidrides are not limited to compounds formed from the dimerisation of C_9_-units. Scytalidin 19 shows antifungal activity with low phytotoxicity, and was first identified due to its fungitoxic effects towards *Poria carbonica*, a wood-rotting fungus.^39^ Recent work has confirmed the absolute and relative configurations of both scytalidin 19 and deoxyscytalidin 20.^8^ In 1989 a ring hydroxylated analogue of scytalidin 19 named castaneiolide 21 was isolated from *Macrophoma castaneicola*, which causes ‘black root rot disease’ in chestnut trees. Assays using the purified castaneiolide 21 showed that it induced wilting in chestnut leaves.^41^ More recent studies have confirmed the structure of castaneiolide 21 (Fig. 4).^8^

The structure of heveadride 22, isolated from *Bipolaris heveae*, was solved in 1973 by MacMillan and co-workers through degradation studies.^42^ Interestingly this nonadride shows a different substitution around the 9-membered ring compared with the byssochlamic acids, scytalidins and rubratoxins and has neighbouring side-chains on the same side of the molecule, reminiscent of glauconic and glaucanic acids 4 and 5, arising from a head-to-head dimerisation. In 1987 a longer chain analogue of 22, homoheveadride 23 was isolated from the lichen symbiont *Cladonia polycarpoides*.^43^

Dihydroepiheveadride 24, a γ-hydroxybutenolide analogue of heveadride 22, as well as epiheveadride 25, were later isolated from an unidentified fungus, with 24 providing significant antifungal activity.^44^ Heveadride 22 and epiheveadride 25 also produced a fungitoxic effect, albeit significantly weaker than dihydroepiheveadride 24.^44^ In 2010 *Wicklowia aquatica* was shown to be a prolific producer of heveadride analogues, producing epiheveadride 25, dihydroepiheveadride 24, deoxoepiheveadride 26, tetrahydroepiheveadride 27, dideoxoepiheveadride 28, and deoxodihydroepiheveadride 29 (Fig. 4).^45^ Of these, 27–29 did not appear to show antifungal activity.^45^ Another heveadride analogue, curvulariahawadride 30 has recently been isolated from a *Curvularia* sp. and was shown to have nitric oxide production inhibitory activity (Fig. 4).^46^

**Fig. 5:**
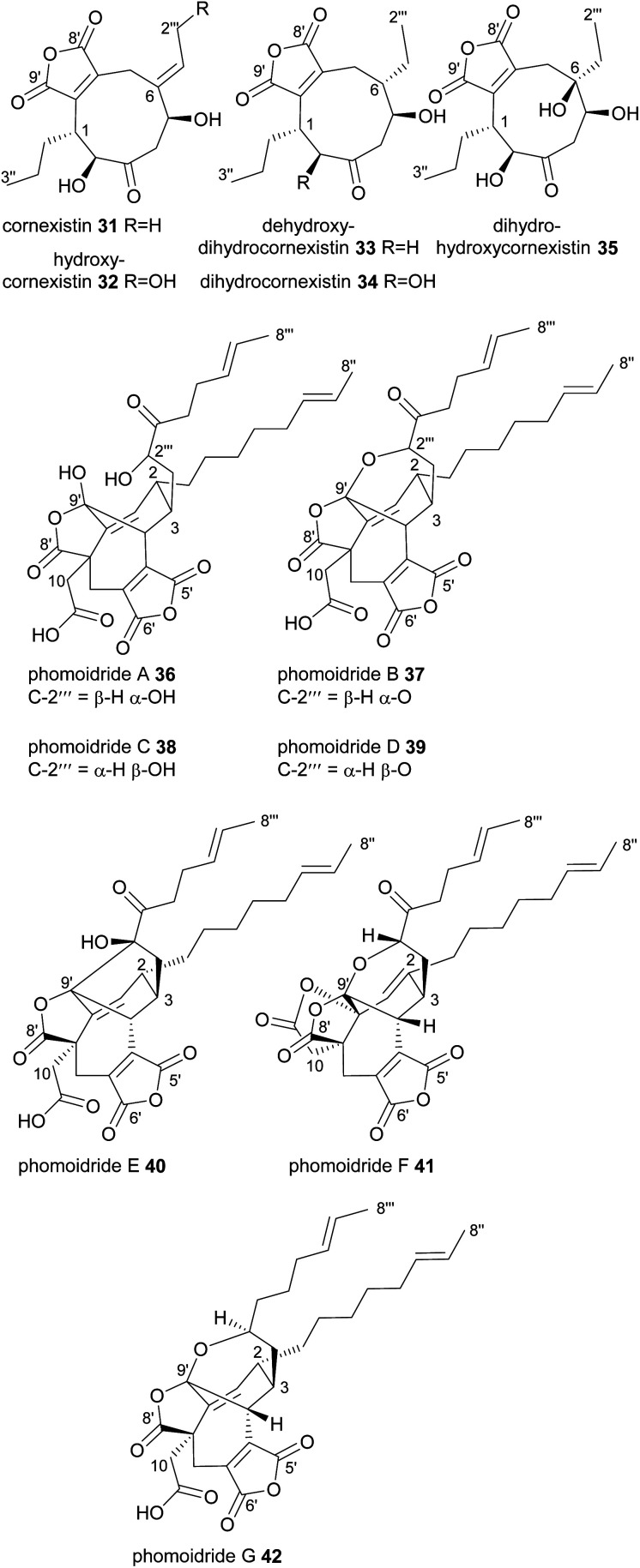
Nonadrides 31–42, with carbons numbered according to the system described in de Mattos-Shipley *et al.*^8^

In contrast to all the nonadrides discussed above, cornexistin 31 and its derivatives contain only one maleic anhydride moiety (Fig. 5). Cornexistin 31 was isolated and characterised in 1992 by the Sankyo pharmaceutical company.^47^ It is produced by the thermotolerant fungus *Paecilomyces divaricatus*, which is closely related to the byssochlamic acid 6 producer, *P. fulvus*.^48^ Cornexistin 31 has significant broad-spectrum phytotoxic activity and is of especial interest due to its low toxicity to the crop plant maize (*Zea mays*).^47^ It also appears to have a unique mode of action, possibly involving inhibition of the plant aspartate amino transferase.^35^ A derivative of cornexistin 31, hydroxycornexistin 32, was later isolated from *P. divaricatus*, which has significantly stronger activity against broadleaf weeds.^49^ Intermediates 33, 34 and 35 from the cornexistin biosynthetic pathway were later isolated from a *P. divaricatus* strain engineered to produce fewer competing metabolites, thus allowing for greater flux towards the cornexistin pathway.^50^

In 1997 the phomoidrides A 36 and B 37 were isolated from cultures of a fungus (ATCC 74256), later identified as belonging to the pleosporales order.^51–53^ Trace amounts of an epimer, phomoidride D 38 were also isolated.^51,52,54^ The phomoidrides A 36 and B 37 have been shown *in vitro* to inhibit squalene synthase and Ras farnesyl transferase and therefore are attractive lead structures for the development of both cholesterol lowering and anticancer drugs.^51^ A further isomer named phomoidride C 39 was isolated in 2001.^55^ Recently, three further phomoidrides have been isolated from ATCC 74256, phomoidrides E 40, G 41 and F 42 (Fig. 5).^53^ The phomoidrides are nonadrides assembled on a complex central core with functionalised side chains at C-2 and C-3. It is apparent however that they are formed from a head-to-head dimerisation in a manner somewhat similar to the glauconic and glaucanic acids 4 and 5. They are unique amongst the maleidrides discovered thus far in that the carboxylic acid of one of the monomers appears to be retained in the mature structure. This is corroborated by feeding studies which demonstrate that the C-10 carboxylic acid is derived from succinate.^56^

**Fig. 6:**
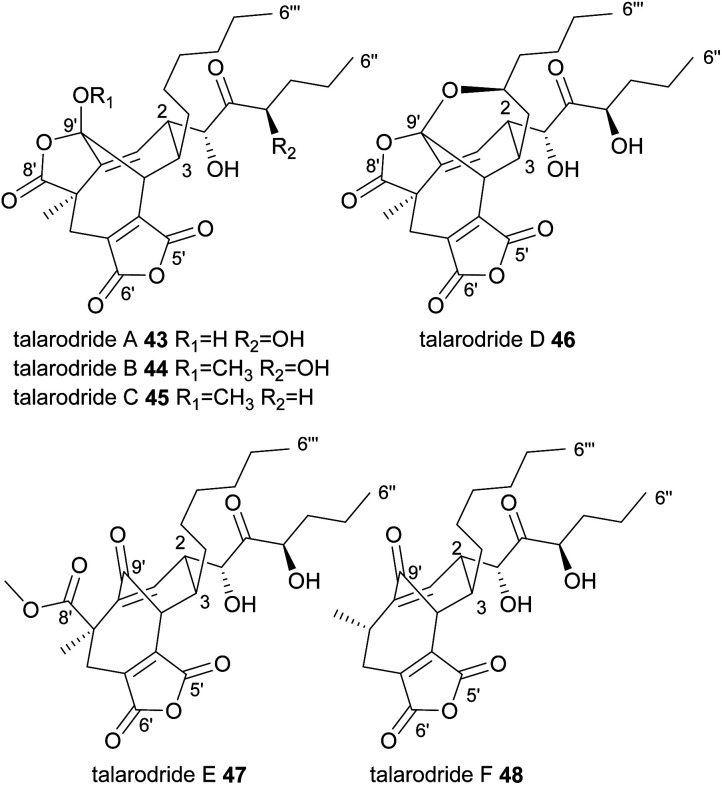
Nonadrides 43–48, with carbons numbered according to the system described in de Mattos-Shipley *et al.*^8^

Very recently, six further nonadrides, the talarodrides A–F 43–48 were isolated from an Antarctic sponge derived fungus, *Talaromyces* sp. HDN1820200 (Fig. 6).^57^ These unusual maleidrides also appear to be formed in a similar manner to glauconic and glaucanic acids 4 and 5, and share the bridgehead olefin present in most phomoidrides *e.g.*37. Talarodrides A 43 and B 44 show specific antibacterial activity against *Proteus mirabilis* and *Vibrio parahemolyticus*.^57^ The methoxy groups present in talarodrides B 44 and C 45 are potentially artefacts due to the use of methanol during isolation.^57^

The structures of the nonadrides have attracted significant attention from the scientific community not only because of their fascinating biosynthesis but also their structures have proved a challenge to the skills of synthetic chemists. Stork completed the first total synthesis of racemic byssochlamic acid in 1972 (ref. 58) and was later followed by White's “photoaddition–cyclodimerisation” strategy for the efficient assembly of the functionalised 9-membered ring.^59^ The first enantioselective synthesis was reported by White and co-workers in 2000 following a similar approach used in the synthesis of the racemate.^60^ The molecular complexity of the phomoidrides has demanded the development of selective strategies and several elegant total syntheses have been achieved.^61–64^ Cornexistin 31 and related compounds have been of particular recent interest due to their potential value as herbicides.^47,49^ Clark and Taylor^65–67^ have explored synthetic routes towards cornexistin 31 and in 2020 the first total synthesis of (+)-cornexistin was reported by Magauer and co-workers.^68,69^ Starting from malic acid, key steps included a Hiyama–Kishi coupling, stereoselective aldol reaction and intramolecular alkylation to deliver >150 mg of cornexistin 31. This approach could be readily adapted for the preparation of analogues.

### 2.2. Octadrides

**Fig. 7:**
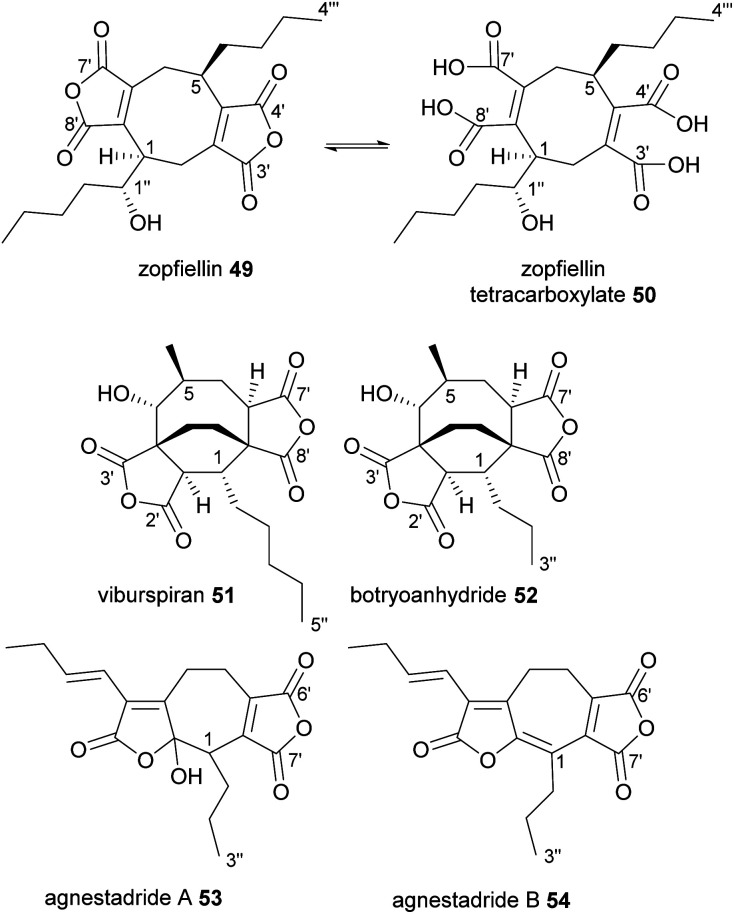
Octadrides 49–52, and heptadrides 53 and 54, with carbons numbered according to the system described in de Mattos-Shipley *et al.*^8^

Zopfiellin 49 was the first octadride to be reported, and was isolated from *Zopfiella curvata* in 1994, by Nissan Chemical Corp.^70^ It shows promising antifungal activity against many plant pathogenic fungi, as well as various fungi that cause human diseases.^70^ Zopfiellin 49 readily interconverts between the ring-closed dianhydride form and the ring-open tetracarboxylate 50, which is favoured at low pH (Fig. 7).^36^ The dianhydride form does not appear to have significant fungicidal activity.^36,71^ The activity of zopfiellin 49/50 is ameliorated by addition of oxaloacetate to fungal cultures, suggesting that the mode of action is associated with oxaloacetate metabolism.^36^ Zopfiellin 49 was recently isolated from a close relative of *Z. curvata*, *Diffractella curvata*, and using a combination of NMR spectroscopy and the X-ray structure of a crystalline derivative, the absolute and relative configurations of zopfiellin 49 were confirmed.^8^

Another antifungal octadride, viburspiran 51, was isolated from *Cryptosporiopsis* sp. in 2011.^72^ Viburspiran 51 contains an ethylene bridge between C-3 and C-8. A similar metabolite, botryoanhydride 52, was recently isolated from an uncharacterised fungus which has an *n*-propyl group attached to C-1, instead of the *n*-pentyl group present in viburspiran (Fig. 7).^73^

### 2.3. Heptadrides

The first natural heptadrides, agnestadrides A 53 and B 54, were isolated from the byssochlamic acid 6 producer, *P. fulvus* in 2015 (Fig. 7).^1^ Baldwin and co-workers had previously characterised a compound with a heptadride structure during their biomimetic investigations into nonadride monomer dimerisation.^74^ A head-to-side mode of dimerisation can explain the formation of the seven-membered central carbocycle (see Fig. 1 and Section 4.2 for more detail).^1,75^

## 3. Origin of the monomers

Soon after the first structure elucidation of the maleidrides, Sutherland and co-workers^5,11^ proposed that their biosynthesis may proceed *via* the coupling of two monomeric units. They were prescient in their hypotheses, proposing that monomer units could be derived from a citric acid intermediate, and that an anionic type coupling mechanism in either head-to-head or head-to-tail coupling could account for the structural differences between glauconic and glaucanic acids 4 and 5, and byssochlamic acid 6.^5^

**Scheme 1:**
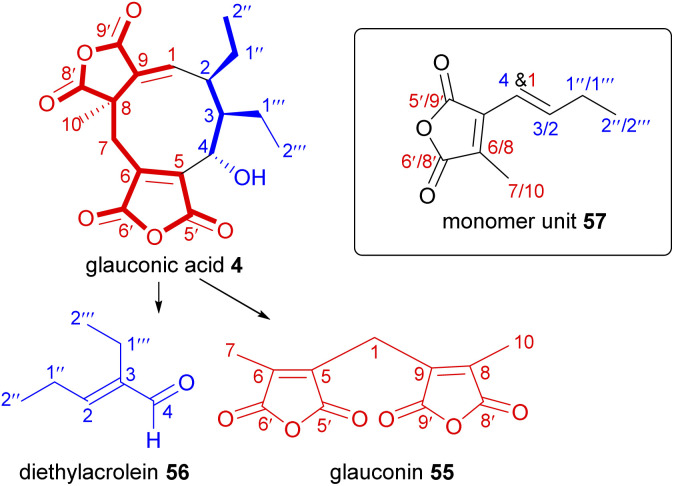
Pyrolytic degradation route of glauconic acid 4, with positions of the equivalent and distinguishable carbons identified with reference to the putative monomer unit 57.

To investigate the biosynthetic construction of the putative monomers, Sutherland and co-workers^76^ performed a series of feeding experiments with ^14^C-labelled putative biosynthetic precursors combined with degradation studies. As the degradation of glauconic acid 4 into characteristic fragments had been previously established,^5,9^ Sutherland and co-workers^76^ selected 4 for these studies as it would undergo controlled decomposition to two known products: glauconin 55 and diethylacrolein 56, and then further degraded to CO_2_ and the radioactivity measured (Scheme 1). The identified carbons could then be referenced to the putative monomer unit, 57.

**Scheme 2:**
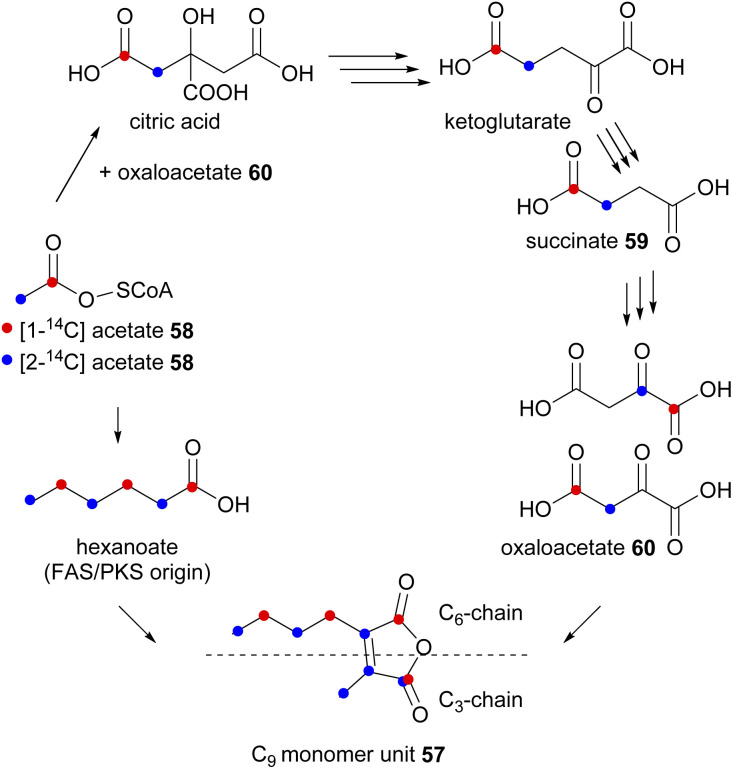
Proposed route of incorporation of labelled acetate into the glauconic acid 4 C9 monomer unit 57*via* the citric acid cycle and the activity of an FAS/PKS.^76,77^

In an initial experiment, a *P. purpurogenum* culture was fed separately [1-^14^C]- and [2-^14^C]-acetate 58, subsequently, labelled glauconic acid 4 was isolated (with 9.4% and 13.2% incorporation radiolabel respectively) and the site of isotopic labelling determined by degradation studies as shown in Schemes 1 and 2.^76^ From these experiments it was deduced that the C_9_-precursor 57 was assembled from two different components coupled to generate the double bond of the maleic anhydride (Scheme 2). The observed labelling pattern was consistent with the longer C_6_-chain of the monomer unit being the product of a typical polyketide/fatty acid synthase (PKS/FAS), derived from a head-to-tail condensation of an acetate and two malonate units (Scheme 2). Two adjacent carbons from the C_3_-chain showed similar incorporation of radioactivity from [2-^14^C]-acetate implying that these carbons have become equivalent in a precursor. To account for this, Sutherland and co-workers^76^ proposed that labelled acetate also enters the citric acid cycle (Scheme 2), where it subsequently labels the truly symmetrical intermediate, succinate 59. Succinate 59 is then converted to oxaloacetate 60, where the [2-^14^C]-acetate 58 activity is distributed equally between the methylene and carbonyl groups.^76,77^

The above experiments^76^ were supported by feeding [2,3-^14^C_2_]-succinate 59, which was observed to be efficiently incorporated into the C_3_-chain. The authors concluded that oxaloacetate 60 is the likely direct precursor of the C_3_ chain.^76,77^

A complementary experiment was undertaken by Cox and Holker with [2,3-^13^C_2_]-succinate 59 fed to *P. purpurogenum*^78^ confirming that intact succinate 59 (or its derivative) was incorporated into the C_3_-chain of the glauconic acid 4 precursor.^78^ Further evidence for the biosynthetic origin of the monomers came from feeding studies using the rubratoxin producer *P. rubrum*. Analysis of the isolated rubratoxin B 11 revealed a labelling pattern in accordance with the longer chain (here C_10_) being derived from a fatty acid and the shorter C_3_ from the citric acid cycle.^79^

**Scheme 3:**
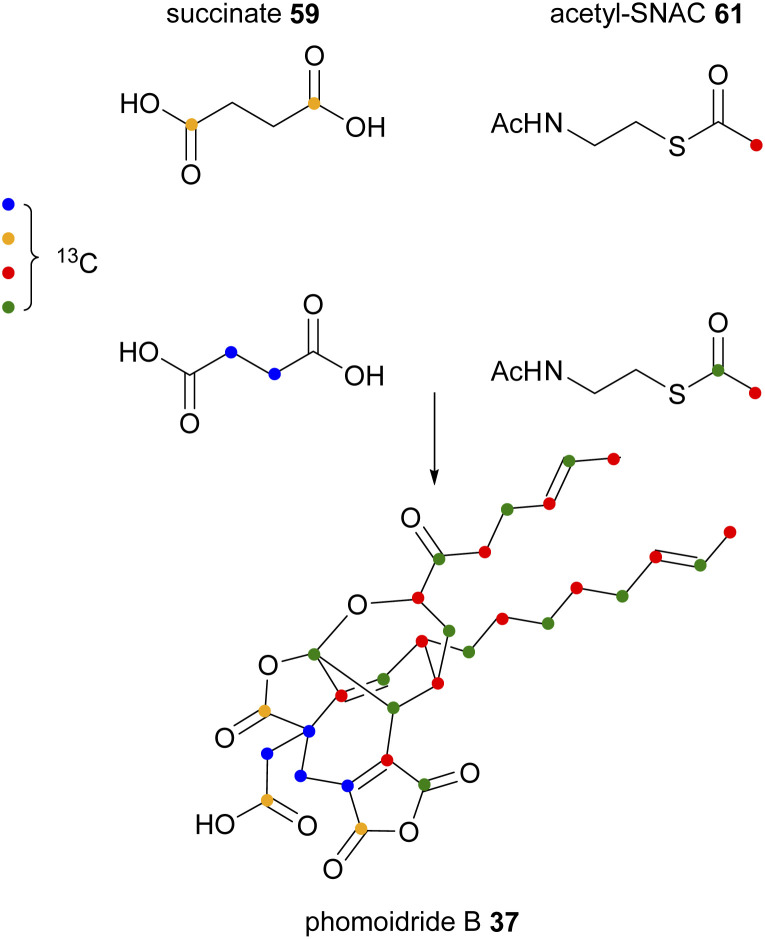
Incorporation of various labelled precursors into phomoidride B 37.^56^

The origin of the putative monomers that form phomoidride B 37 has also been investigated.^56^ The producing organism, unidentified fungus ATCC 74256, was fed a series of carbon-13 labelled precursors, and phomoidride B 37 isolated and analysed by ^13^C NMR. The deduced labelling pattern shown in Scheme 3 was in full accordance with the longer C_12_-chain being derived from a polyketide/fatty acid synthase.

**Fig. 8:**
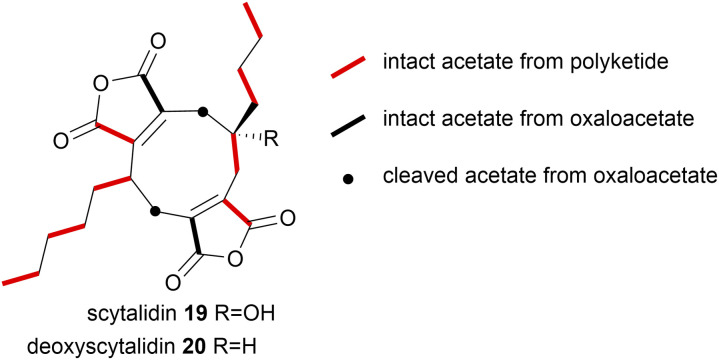
[1,2-^13^C_2_]-Acetate 58 incorporation into scytalidin 19 and deoxyscytalidin 20.^8^

In more recent investigations by Willis and co-workers^8^ on the biosynthesis of the nonadrides scytalidin 19 and deoxyscytalidin 20, [1,2-^13^C_2_]-acetate 58 was fed to cultures of *S. album* and analysis of the ^13^C-NMR data of both metabolites was in accord with the polyketide and oxaloacetate origin of the natural products (Fig. 8).

## 4. Evidence for dimerisation during maleidride biosynthesis

**Scheme 4:**
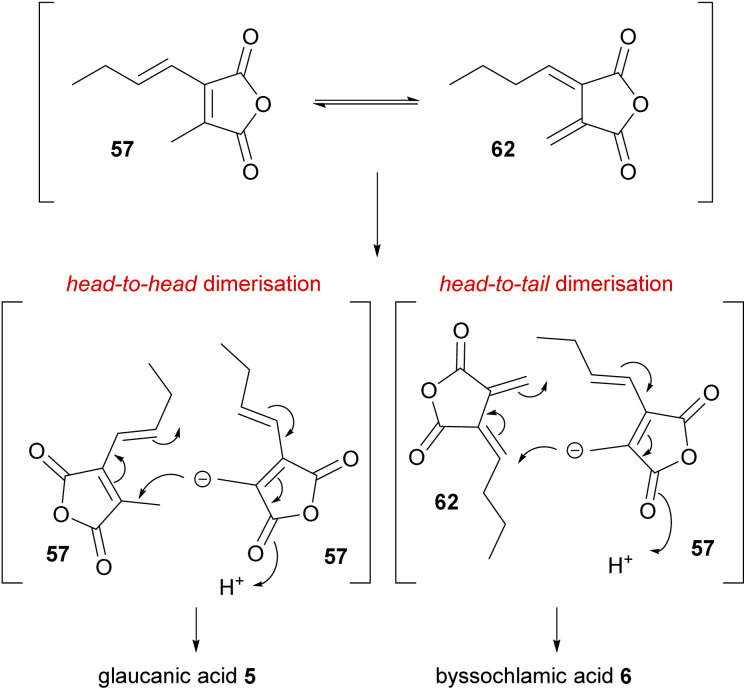
Mechanisms uniting the biosyntheses of glaucanic acid 5 and byssochlamic acid 6 according to Barton and Sutherland.^5^

As discussed in Section 3, in 1965 Barton and Sutherland^5^ with immense prescience had proposed that the biosyntheses of glauconic and glaucanic acids 4 and 5, and byssochlamic acid 6 may originate from similar building blocks (monomers) but coupled in different ways to generate the various carbon skeletons. The head-to-head anionic coupling mechanism proposed for the biosynthesis of glauconic and glaucanic acids 4 and 5, requires two identical 57 monomers (Scheme 4). The head-to-tail coupling required for byssochlamic acid 6 biosynthesis would require one monomer 57 and the *exo*-diene analogue 62 (Scheme 4).

**Fig. 9:**
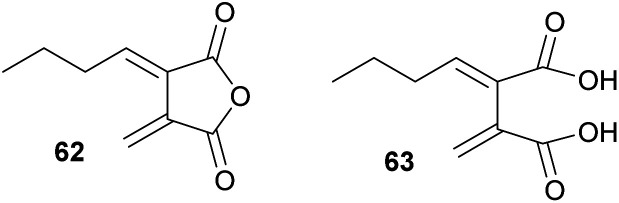
Structural revision of the natural product waquafranone B 62 from to 63 as proposed by Willis and co-workers.^80^

The *exo*-diene 62 (herein named waquafranone B) had been reported to have been isolated from *W. aquatica*, a producer of a variety of heveadride analogues (*e.g.*25).^45^ However, recent biomimetic dimerisation studies by Willis and co-workers^80^ revised the structure of waquafranone B to be diacid 63 (Fig. 9). This is in accord with biomimetic studies by Sutherland and co-workers^81^ who demonstrated that *exo*-diene 62 is unstable.

The instability of the *exo*-diene 62 does not preclude its veracity as a true intermediate in maleidride biosynthesis, as unstable intermediates may be chaperoned by enzymes *in vivo*. The equilibrium represented between 57 and 62 in Scheme 4 is a regiochemical rationalisation depicted to describe a potential enzyme catalysed mechanism that remains to be proven.

In 2000 Sulikowski, Agnelli and Corbett were the first to propose that the maleidride monomer might contain a carboxylic acid, likely due to their specific interest in the phomoidrides, where one carboxylic acid is retained in the mature natural product.^82^ They proposed that the reactive anionic monomer is derived from decarboxylation of monomer 1.

**Scheme 5:**
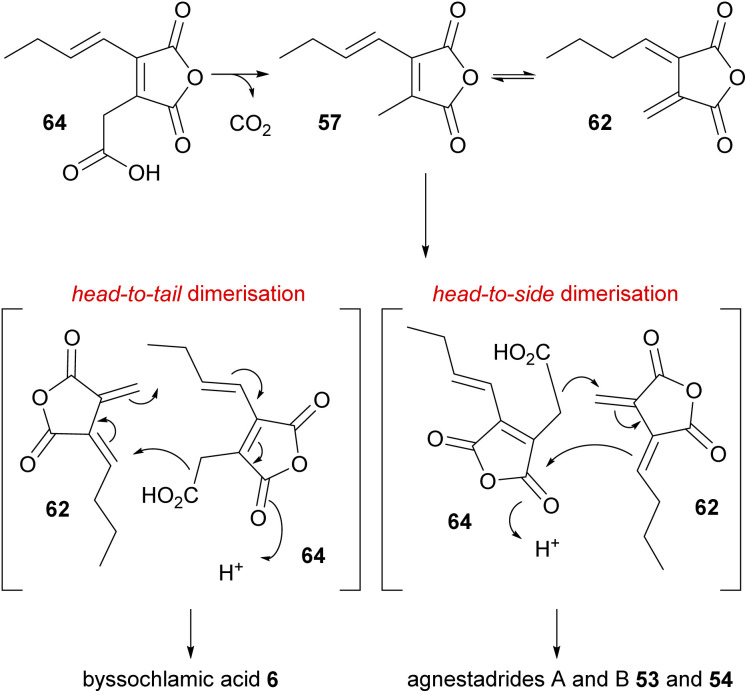
Dimerisation mechanisms proposed for the biosynthesis of byssochlamic acid 6 and agnestadrides A and B 53 and 54*via* the decarboxylation of monomer 64.^1^

Isolation of the carboxylated analogue of the anhydride 57, monomer 64, from the byssochlamic acid 6 producer *P. fulvus*, and the previous feeding studies by Sulikowski and co-workers,^82^ led Simpson and co-workers^1^ to speculate that carboxylated monomer 64 coupled with *exo*-diene 62 may be the true intermediates for byssochlamic acid 6 biosynthesis, as well as for the newly discovered heptadrides 53 and 54 also isolated from *P. fulvus* (Scheme 5). The authors noted that in their hands carboxylated anhydride 64 was unstable, and completely decomposed to 57 in under 48 hours.^1^

Key evidence for the involvement of a dimerisation step during maleidride biosynthesis has come from four sources: (i) feeding experiments performed *in vivo*; (ii) *in vitro* chemical investigations of the substrates, reaction conditions and their products; (iii) from combined chemical and genetic studies in maleidride producers; and (iv) from cell free biocatalysis with the proposed dimerisation enzymes.

### 4.1. *In vivo* studies

**Scheme 6:**
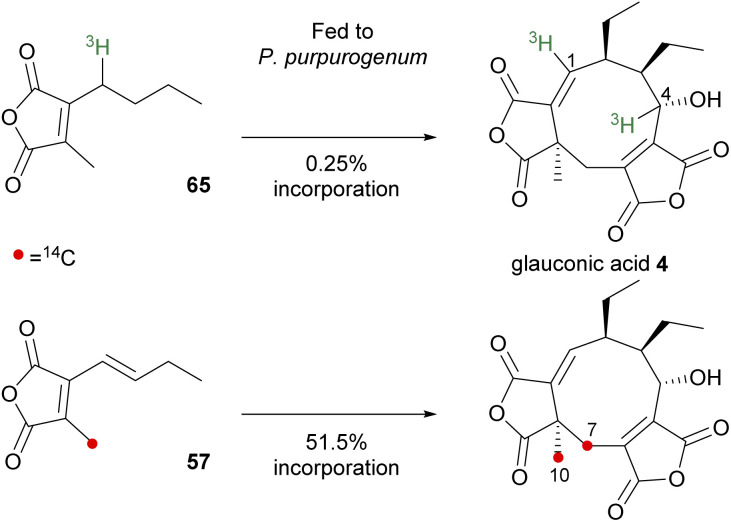
Incorporation of monomer analogues into glauconic acid 4.^83^

The first direct evidence for *in vivo* incorporation of maleic anhydride-based monomers into the structure of a nonadride metabolite was reported for glauconic acid 4 (Scheme 6).^83^ The study by Moppett and Sutherland^83^ involved separately feeding two isotopically labelled substrates, tritiated 65 and carbon-14 labelled 57, into liquid cultures of the glauconic acid 4 producer, *P. purpurogenum*. Feeding compound 65 afforded [1,4-^3^H_2_]-glauconic acid 4 which was confirmed by degradation studies leading to an equal label distribution between glauconin 55 (C-1) and diethylacrolein 56 (C-4) (degradative studies are shown in Scheme 1). A 1 : 1 ratio of activities established that dimerisation had taken place, however the incorporation was very low (0.25%).

Incubating growing cultures of *P. purpurogenum* with the ^14^C-labelled 57 resulted in the isolation of glauconic acid 4 with 51.5% incorporation of carbon-14, with 97.5% of the total activity localised at C-7 and C-10 (Scheme 6).^83^ In both experiments, the radiolabels were found at positions expected for the product of head-to-head dimerisation of the fed monomer units, and the higher level of incorporation of 57 suggested that the unsaturated anhydride is the correct monomer unit.^83^

Sulikowski and co-workers sought a biomimetic approach towards the total synthesis of phomoidrides A 36 and B 37,^56^ and this led the group to pursue biosynthetic studies in the unidentified fungus ATCC 74256 using precursors incorporating stable isotopic labels. Although phomoidrides A 36 and B 37 and glauconic acid 4 differ in the length of the pendant side-chains, the same symmetrical pattern can be discerned and consequently phomoidrides A 36 and B 37 were proposed to be formed through coupling of analogous C_16_-precursor units.^52^

**Scheme 7:**
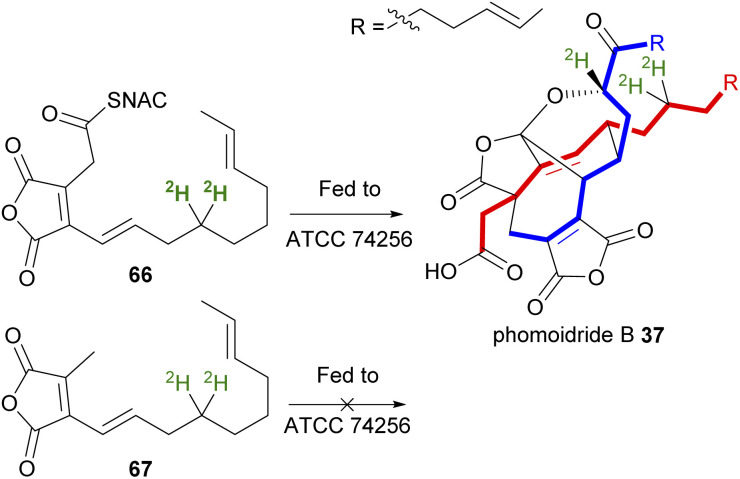
Incorporation of deuterium label into phomoidride B 37*via* a decarboxylative homodimerisation event involving C_16_-monomers.^84^ The two monomer units present in phomoidride B 37 are depicted in red and blue.

Sulikowski and co-workers^84^ prepared synthetic analogues of the predicted precursors incorporating deuterium (Scheme 7). The first synthetic substrate was thiol ester 66, as *N*-acetylcysteamine (SNAC) has been shown to be a valuable CoA substitute in biosynthetic studies, as it can readily pass through cell membranes, unlike CoA adducts. These CoA mimics are often used where carrier protein-bound thioesters are required in enzyme biosynthetic machinery, for example when investigating polyketide biosynthesis.^85^ Sulikowski and co-workers^84^ fed [^2^H_2_]-thiol ester 66 to a culture of ATCC 74256 and phomoidride B 37 was isolated with incorporation of 3 deuterium atoms as determined by ^2^H NMR and ESIMS analysis. This provided evidence for a homodimerisation process having occurred (Scheme 7). A similar experiment with [^2^H_2_]-67, with a pendant methyl group rather than the thiol ester, did not show any incorporation into phomoidride B 37 (Scheme 7). This important experiment provided the first evidence that dimerisation requires decarboxylation, at least in the case of the phomoidrides.^84^

### 4.2. Biomimetic studies

**Scheme 8:**
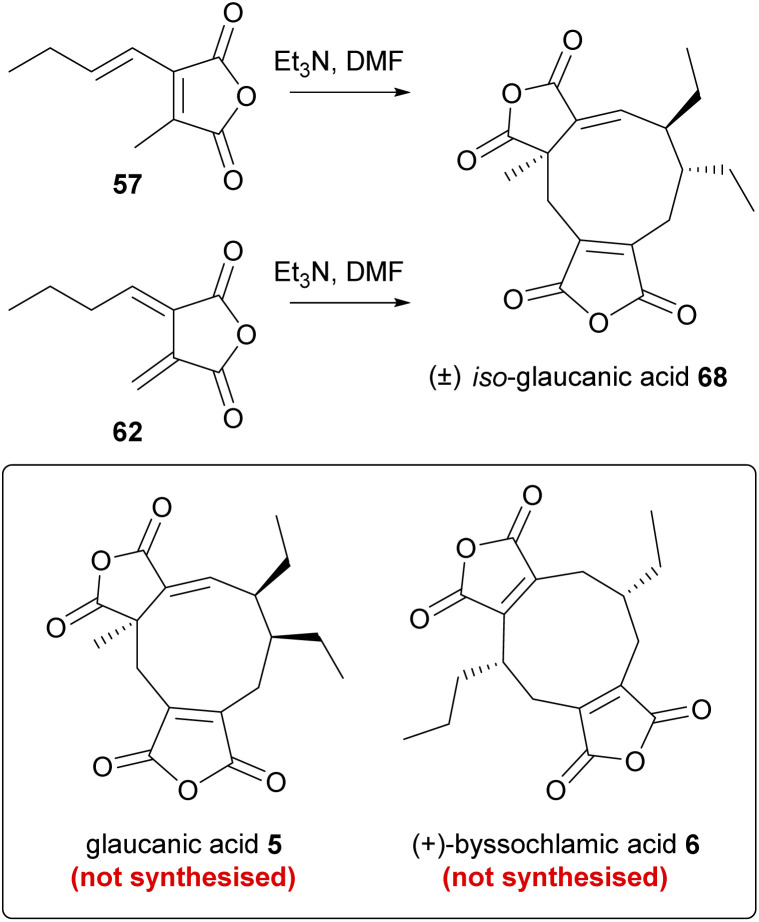
*In vitro* dimerisation reactions investigated by Sutherland and co-workers.^81,86^

Several biomimetic synthetic studies aimed at reconstructing the maleidride dimerisation event under laboratory conditions provide interesting insights into the mechanism of the reaction. Upon completing feeding studies with anhydride 57, Huff, Moppett and Sutherland set out to test self-dimerising properties *in vitro*.^81,86^ To this end, maleic anhydride 57 was treated with base in order to generate the required carbanion intermediate. The reaction afforded a crystalline solid in a very low yield (2% with NaH, improved to 4% by using Et_3_N), which was not the expected glaucanic acid 5, but believed to be iso-glaucanic acid 68, a stereoisomer of the natural product formed *in vivo* (Scheme 8).^86^ In parallel, an attempt was made to synthesise fulgenic anhydride 62, in order to test a hypothesis that this compound might be involved in the reaction leading specifically to the formation of byssochlamic acid 6.^81^ However, the base-catalysed *in vitro* dimerisation reaction of the fulgenic anhydride 62 again yielded iso-glaucanic acid 68 and not byssochlamic acid 6 (Scheme 8). This was rationalised to be due to the instability of anhydride 62, which under the reaction conditions was found to isomerise to 57.

Interest in the dimerisation was reinvigorated almost 30 years later, inspired by the discovery of the phomoidrides^51,52^ and driven by the pursuit of an efficient total synthesis route. The reports on *in vitro* dimerisation came in a series of papers from the groups of Baldwin^74,87^ and Sulikowski,^82,88^ who both set out to investigate the chemical mechanism driving the reaction.

**Scheme 9:**
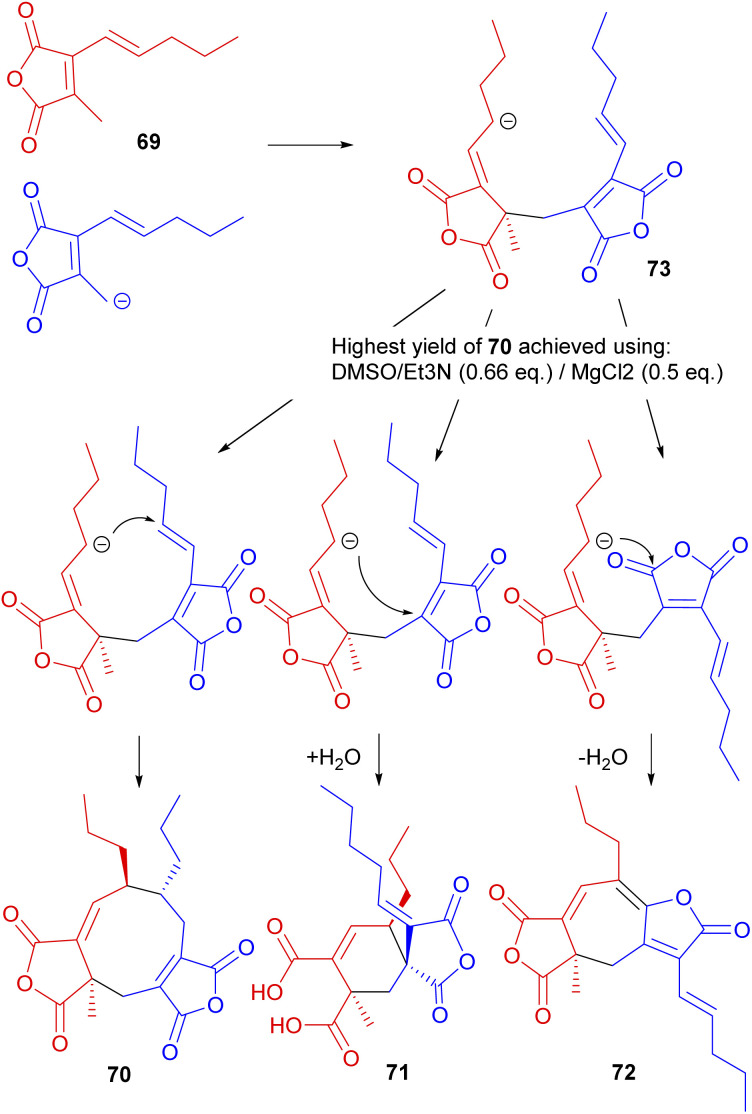
Dimeric compounds formed from biomimetic studies with the anhydride monomer 69.^74^

Studies were reopened by Baldwin and co-workers,^74^ who reinvestigated the *in vitro* dimerisation studies towards glaucanic acid 5.^81,86^ Beside obvious differences in the lengths of the side-chains (and consequently in the structure of the dimerising monomer), there are key differences in the stereochemistry between iso-glaucanic acid 68 and the phomoidrides. Despite this the authors viewed this biomimetic dimerisation as a potential synthetic route towards the phomoidrides.^74^ Thus, 2-[(*E*)-1′-pentyl]-methyl maleic anhydride 69 was synthesised and treated with base under a range of conditions. Although mostly polymeric products were formed, iso-glaucanic acid analogue 70 together with two other minor dimerisation products, the spiro compound 71, as well as the heptadride 72 were isolated in low yields (Scheme 9). A common structural feature of all three products is the linkage of the two anhydride moieties *via* a CH_2_ bridge. Hence Baldwin and co-workers^74^ proposed that a stepwise Michael addition is more likely than a concerted 6π + 4π cycloaddition. Furthermore they suggest that the anion in intermediate 73 is able to attack at different electrophilic centres, accounting for the formation of the different products.^74^

Further optimisation of the reaction conditions was carried out, with the highest yield (8.5%) of 70 achieved using DMSO/Et_3_N (0.66 eq.)/MgCl_2_ (0.5 eq.). X-ray crystallography confirmed the relative stereochemistry of the side-chains in accord with Sutherland's assignment of the configuration of iso-glaucanic acid 68.^86^

In 2000 Sulikowski, Agnelli and Corbett investigating the *in vitro* dimerisation of phomoidride precursors^82^ proposed that within an *in vivo* system at least one of the dimerising units is likely to be covalently linked to an enzyme so imposing conformation constraints. Furthermore, if the dimerisation process is stepwise rather than concerted, *in vitro* studies linking the two monomers prior to cyclisation may lead to cleaner reactions.

**Scheme 10:**
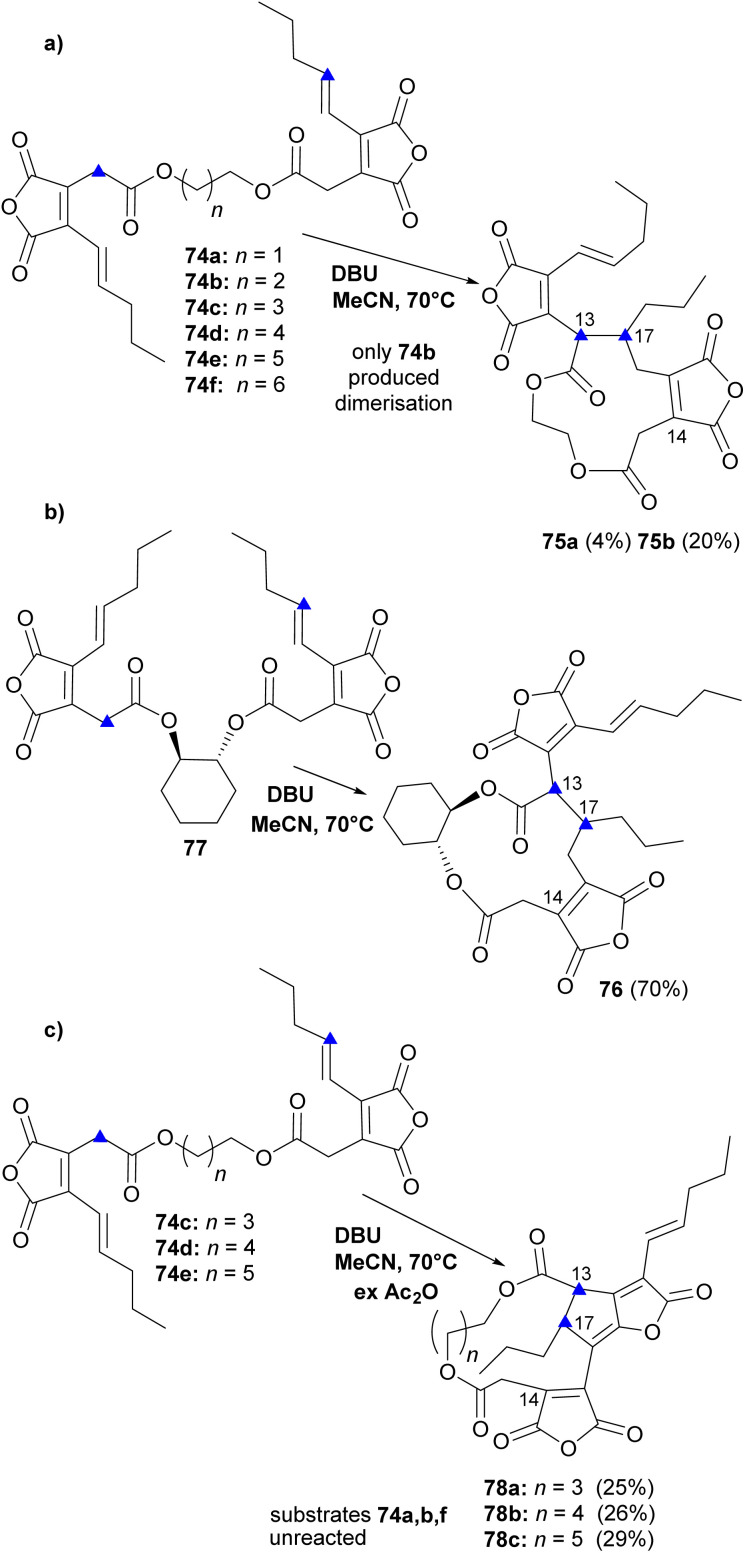
Overview of initial ‘tethered’ *in vitro* dimerisation experiments by Sulikowski, Agnelli and Corbett.^82^ Blue triangles indicate where the formation of the ring closing C–C bond occurred.

In an initial experiment, Sulikowski and co-workers^82^ covalently linked the two units as bis-esters with varying chain-lengths (compounds 74a–f, Scheme 10). Treating a mixture of the six substrates, 74a–f (Scheme 10a, *n* = 1–6) with DBU in anhydrous MeCN triggered dimerisation with only substrate 74b (*n* = 2), to produce 75a and 75b (different stereoisomers at the newly formed stereocentres C-13 and C-17). A single stereoisomer 76 was obtained in an analogous reaction with symmetric diol 77 (Scheme 10b). A mechanism involving a Michael addition was proposed and it was assumed that the observed compounds were the thermodynamic products of the reaction. To trap kinetic products, the reaction using substrates 74a–f was repeated in the presence of excess acetic anhydride (Scheme 10c). Three additional dehydrated products 78a–c were identified, which were derived from substrates 74c–e (Scheme 10c). The position desired for the biomimetic synthesis of phomoidrides requires formation of C-13, C-14 bond. To the authors' disappointment, in all the *in vitro* products, the ring-closing C–C bond was formed exclusively between C-13 of the enolate and C-17 of the Michael acceptor instead.^82^

**Scheme 11:**
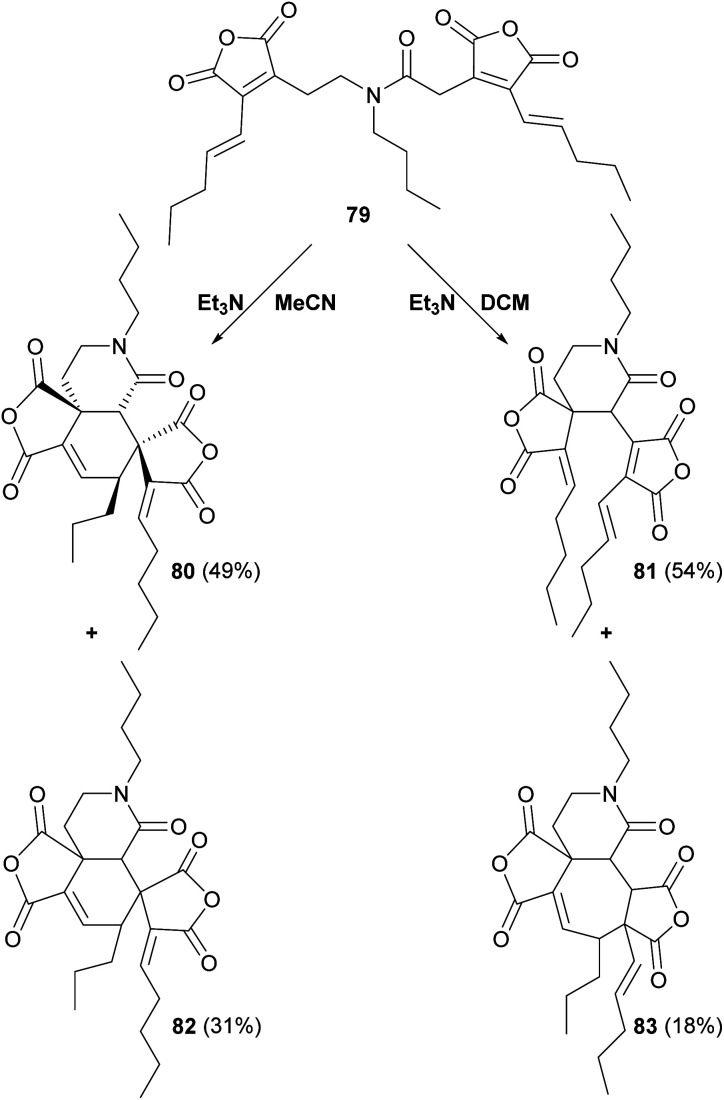
Overview of further ‘tethered’ *in vitro* dimerisation experiments by Sulikowski and co-workers.^88^

Sulikowski and co-workers^88^ modified the substrate by using a tertiary amide linker, to produce substrate 79 (Scheme 11). Whilst products 80, 81, 82 and 83 were formed, no products with the desired phomoidride core were detected.^88,89^

**Scheme 12:**
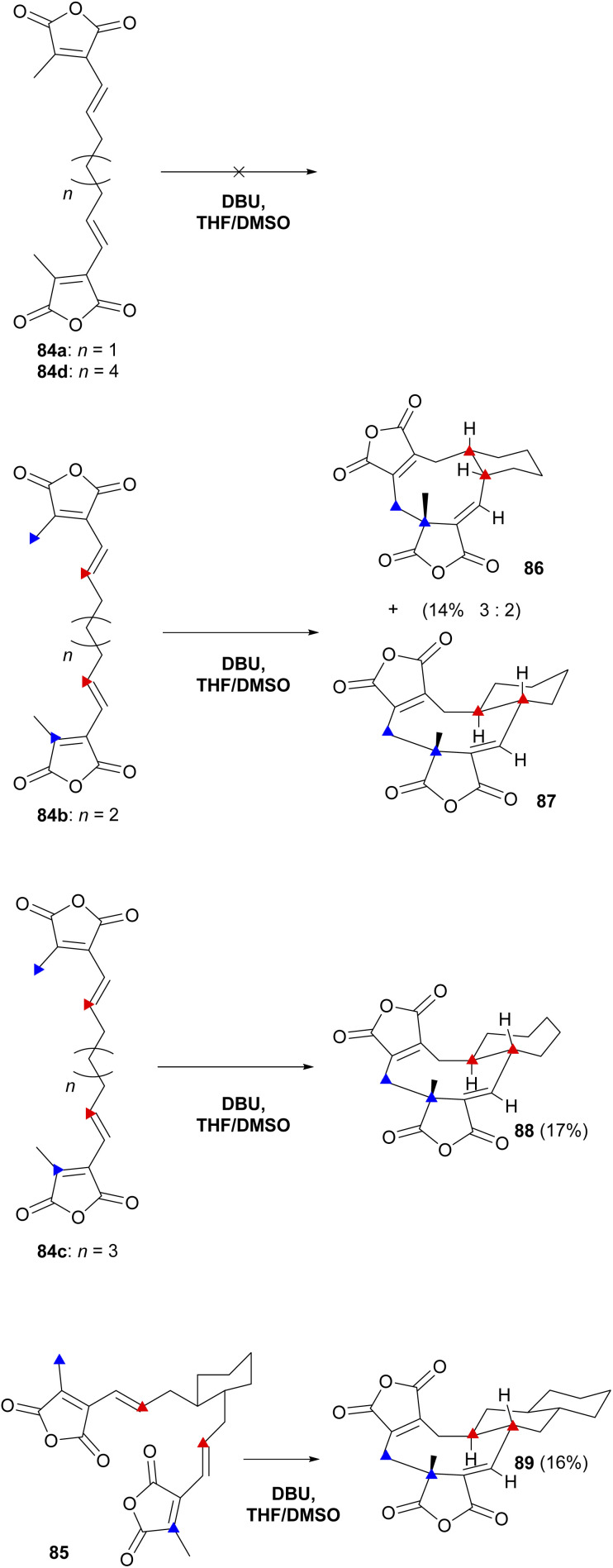
Overview of ‘tethered’ *in vitro* dimerisation experiments by Baldwin and co-workers.^87^ Blue triangles denote bond formation at the free ends of the substrate, red triangles denote the intramolecular bond formation.

Baldwin and co-workers^87^ also investigated the influence of a covalent tether on the stereo- and regioselectivity of cyclisation. Substrates (84a–d and 85) were exposed to a range of reaction conditions and DBU in THF : DMSO (1 : 4) led to cyclisation (Scheme 12). Only three out of the five prepared substrates, 84b, 84c and 85, gave products which could be isolated and characterised showing the structures to be 86, 87, 88 and 89 (Scheme 12). The authors proposed that these cyclic products were the result of *exo*-orientated double Michael additions.^87^

**Scheme 13:**
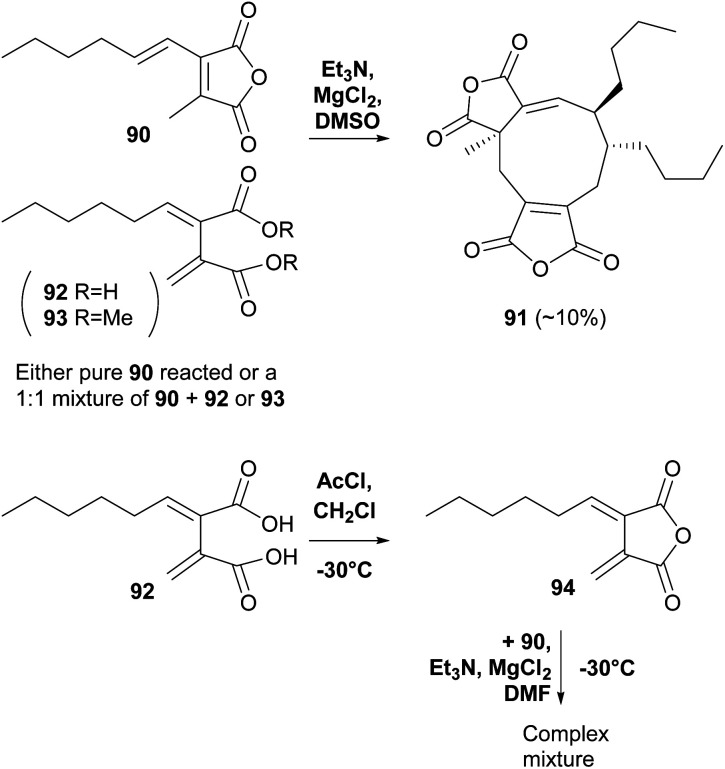
Biomimetic *in vitro* dimerisation studies by Willis and co-workers.^80^

A recent study by Willis and co-workers^80^ into maleic anhydride and related diacid natural products used a biomimetic approach to investigate *in vitro* dimerisations of the proposed monomers required for scytalidin 19 biosynthesis. The authors noted that in all previous biomimetic studies, the focus has been on homodimerisation of analogues of 57, rather than heterodimerisation using 57 and the *exo*-diene 62, which is proposed to be involved in maleidride biosynthesis during various modes of dimerisation (see Schemes 4, 5 and 17 and Section 5). However *exo*-diene 62 was unstable even when kept at −78 °C and after 96 h was converted to a mixture of products including the corresponding maleic anhydride 57. Homodimerisation of the maleic anhydride tetraketide monomer 90 using Et_3_N, MgCl_2_ in DMSO (as used by Baldwin and co-workers^74^) gave iso-glaucanic acid analogue 91 in 10% yield. However, efforts to heterodimerise 90 with either 92 or 93 (avoiding the unstable *exo*-diene), gave iso-glaucanic acid analogue 91 in similar yields, with 92 and 93 recovered from the reaction unchanged. Use of freshly prepared *exo*-diene 94 in a heterodimerisation reaction with maleic anhydride 90 led to a complex mixture of products, none of which could be characterised (Scheme 13).^80^

## 5. Molecular reconstruction of maleidride biosynthesis

### 5.1. Core genes for monomer biosynthesis

**Scheme 14:**
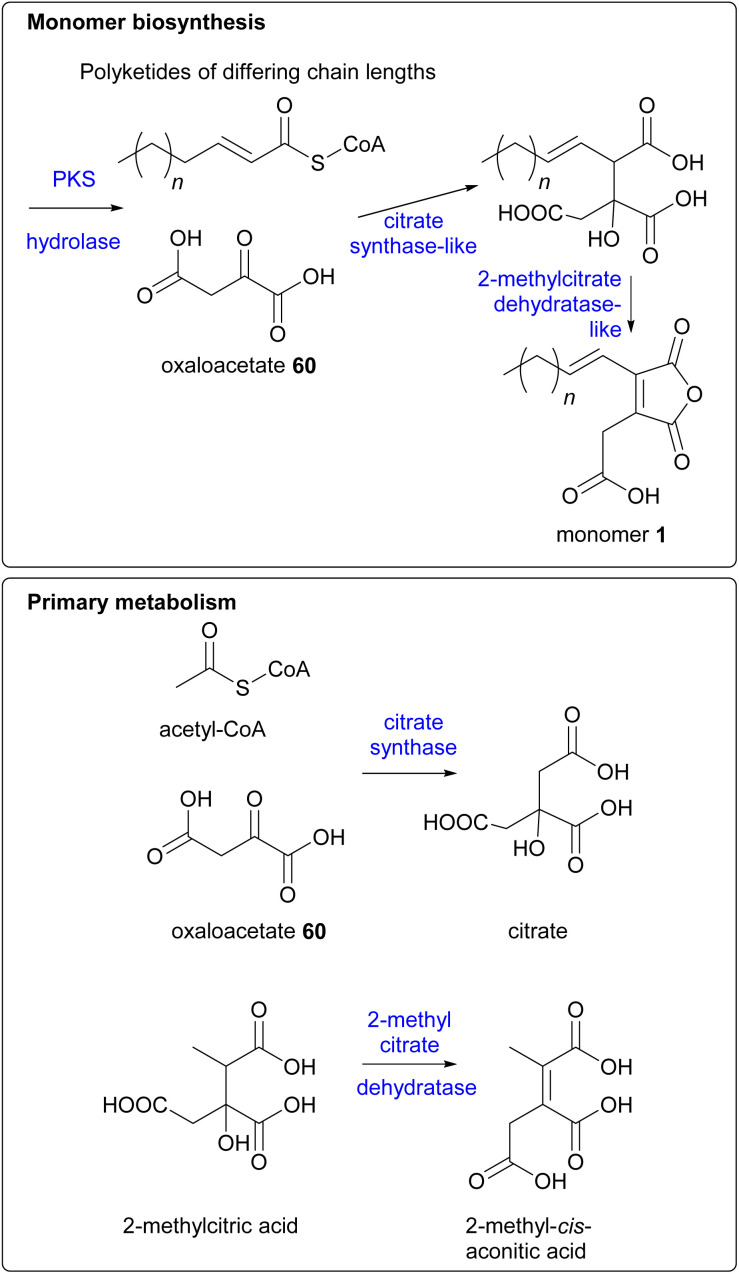
Proposed similarities between the enzymatic reactions in maleidride monomer biosynthesis and primary metabolism.

**Fig. 10:**
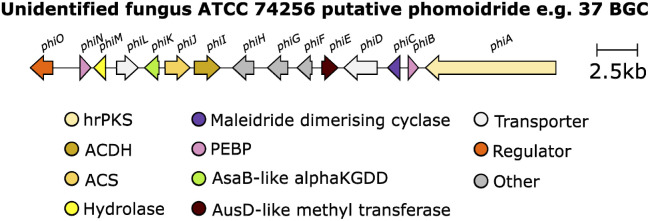
Putative phomoidride *e.g.*37 BGC.

The genetic and enzymatic basis of maleidride biosynthesis remained cryptic until 2015, when Oikawa and co-workers^90^ investigated the biosynthetic pathway for the production of maleidride monomers. In fungi the genes required for the biosynthesis, regulation and transport of a specific natural product are generally co-located as a single biosynthetic gene cluster (BGC).^91,92^ Therefore Oikawa and co-workers^90^ initially sequenced the genome of the phomoidride (*e.g.*37) producer, the unidentified fungus, ATCC 74256, to identify a putative BGC for the production of the phomoidrides (*e.g.*37). As previous feeding studies had demonstrated,^56,76,78^ the likely origin of the maleidride monomer is the condensation of the product of a FAS/PKS with oxaloacetate. Oikawa and co-workers^90^ proposed that a putative maleidride BGC might contain either an FAS/PKS clustered with a gene encoding a citrate synthase-like (CS) enzyme (Scheme 14). They identified a BGC they named *phi* (Fig. 10) which consisted of a highly-reducing PKS (hrPKS), *phiA*, clustered with *phiI*, a gene encoding a CS-like enzyme,^93^ as well as a gene encoding a 2-methylcitrate dehydratase-like enzyme (2MCD, *phiJ*),^94^ which is a likely candidate for the dehydration reaction required to form the unsaturated monomer 1 (Scheme 14). At the time, no genes encoding hydrolytic enzymes for hydrolysis of ACP-bound polyketide chains were detected, although more recent analysis has determined that *phiM* encodes a hydrolase, which is a homologue of the esterase from the asperlin BGC (alnB – C8VJR6.1).^95,96^

Phylogenetic analysis of citrate synthase-like and 2-methylcitrate dehydratase-like enzymes from the likely phomoidride BGC, along with other subsequently discovered maleidride homologues, has determined that these enzymes form a separate clade with those that are known and predicted to produce or accept alkylcitrate.^96^ It is therefore accepted that these enzymes should be referred to as alkylcitrate synthases (ACSs) and alkylcitrate dehydratases (ACDHs).^96^

**Scheme 15:**
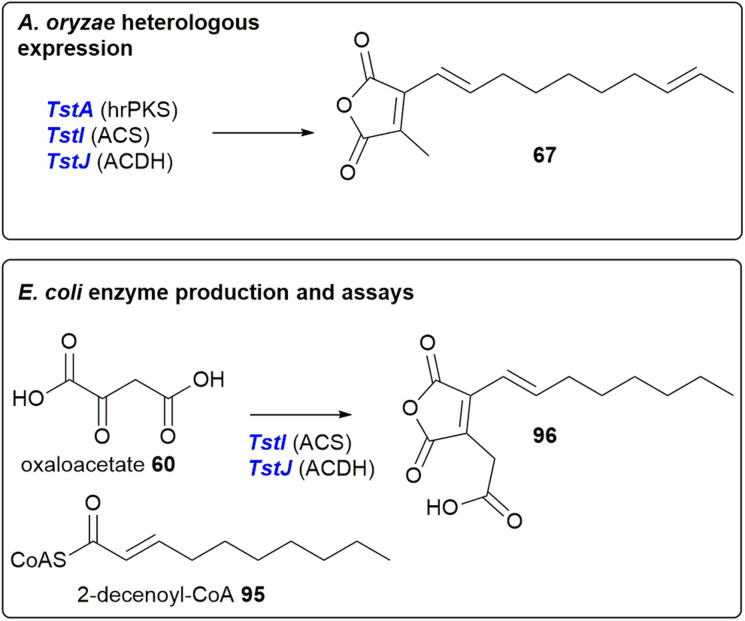
Result of expression of *T. stipitatus* maleidride genes in *A. oyzae*, and enzyme production and assays, conducted by Oikawa and co-workers.^90^

Oikawa and co-workers^90^ reconstructed *phiA*, *I*, *J* in the heterologous host *Aspergillus oryzae* (a suitable host for the production of fungal natural products).^97–99^ This resulted in the production of a new metabolite which possessed the characteristic UV absorption (*λ*_max_ 312 nm) for a maleic anhydride conjugated with an olefin.^90^ Due to low titres, no specific product of *phiA*, *I*, *J*, was isolated and so the attention of the authors turned to a homologous cluster, *tst*, which they had identified in the publicly available *Talaromyces stipitatus* genome. Although *T. stipitatus* itself has not been reported to produce maleidrides, many *Talaromyces* species are known to produce glauconic and glaucanic acids 4 and 5, as well as the more complex rubratoxins *e.g.*10 (although no *Talaromyces* species are reported to produce phomoidrides).^100^ Expression of the *phiA*, *I*, *J* homologues, *tstA*, *I*, *J* in *A. oryzae* resulted in the production of a compound with similar LCMS characteristics to that which was produced by the heterologous expression of *phiA*, *I*, *J*. The structure was confirmed to be 67 by NMR and HRMS (Scheme 15). Compound 67 is the predicted monomer required for phomoidride biosynthesis, and is an analogue of the substrate 66 successfully utilised in the phomoidride feeding studies conducted by Sulikowski and co-workers (Scheme 7).^84^

Further evidence for the relatedness of the *phi* and *tst* BGCs comes from phylogenetic analyses by Williams *et al.*^96^ This work showed that maleidride PKSs appear to clade according to the expected or confirmed chain length of their polyketide product, with *PhiA* and *TstA* forming a separate ‘hexaketide’ producing clade, which suggests that the *T. stipitatus* cluster may encode phomoidride biosynthesis or a related analogue formed from hexaketide based monomers.^96^

Oikawa and co-workers^90^ also expressed the *tstI*, *J* genes in *Escherichia coli*, followed by purification and enzyme assays utilising 2-decenoyl-CoA 95 and oxaloacetate 60 as substrates. This assay produced compound 96, which is carboxylated, with the polyketide derived moiety one acetate unit shorter than the compound isolated from *A. oryzae* (Scheme 15). Details of any further substrates tested were not available, therefore it is difficult to determine if 2-decenoyl-CoA 95 is the true substrate for *TstI* (the alkylcitrate synthase), or whether *TstI* may have some substrate flexibility regarding chain length.

**Scheme 16:**
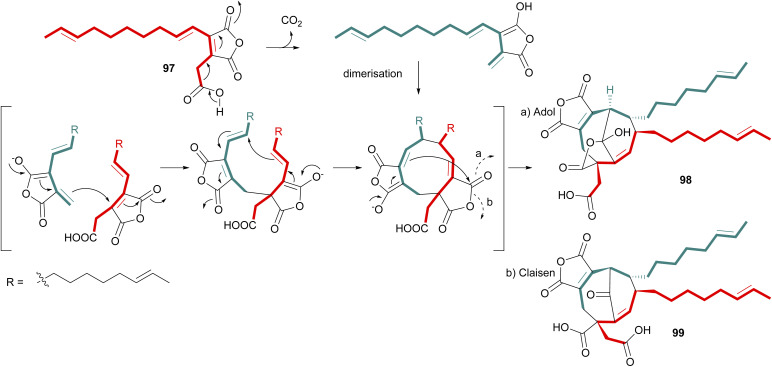
Proposed dimerisation of carboxylated monomer 97 to produce predicted phomoidride intermediate 98*via* an adol-like reaction,^90^ or *via* a Claisen condensation to produce intermediate 99.^53^

Following isolation of the carboxylated monomer 96 from the enzyme assays conducted by Oikawa and co-workers,^90^ (Scheme 15) a mechanism was proposed for dimerisation of a carboxylated analogue of compound 67 (97) to produce the predicted phomoidride intermediate 98*via* an aldol like reaction (Scheme 16). Hu and co-workers^53^ recently isolated further phomoidrides E 40, F 41, and G 42 which led them to propose that the key phomoidride intermediate 99 is more likely to be formed *via* a Claisen condensation (Scheme 16).

**Scheme 17:**
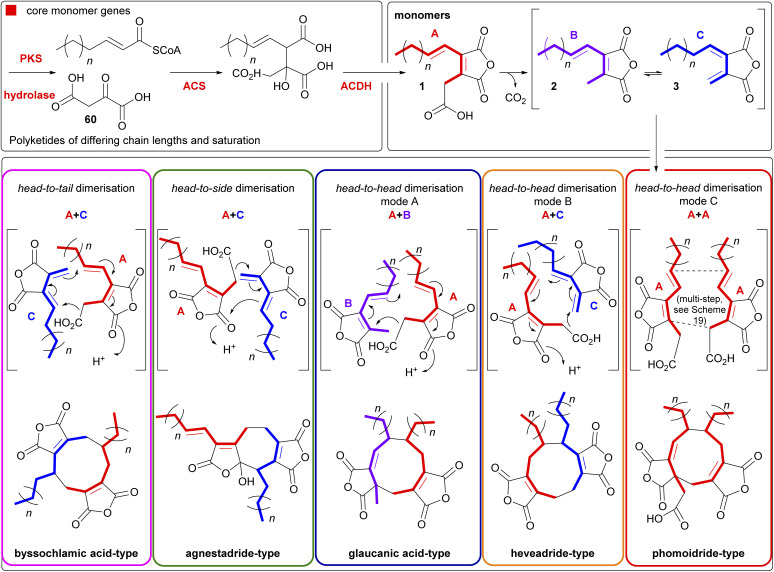
Proposal for a unified pathway to maleidrides driven by enolate formation based on work by Oikawa and co-workers,^90^ and Cox and co-workers.^1,75^ Figure reproduced from ref. 96.

Oikawa and co-workers^90^ have proposed a unified model for maleidride biosynthesis (Scheme 17). This model is based on the homo- and hetero-dimerisations of the carboxylated anhydride, ‘monomer A’ 1, the decarboxylated anhydride ‘monomer B’ 2 and the *exo*-diene anhydride ‘monomer C’ 3, and is driven by the formation of an enolate derived from A 1. The authors proposed that their model accounts for discrepancies in previous feeding experiments, as these appeared to be based on a single monomer.

**Fig. 11:**
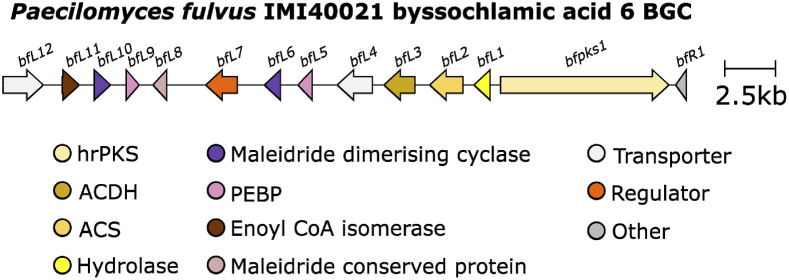
Byssochlamic acid 6 BGC.

In 2016 Cox and co-workers^75^ reported the results of studies on maleidride biosynthesis *via* heterologous expression in the host *A. oryzae*. This study further characterised the pathway for byssochlamic acid 6 and agnestadrides A and B 53 and 54 following on from earlier predictions by Simpson and co-workers.^1^ Genes homologous to those identified by Oikawa and co-workers^90^ (encoding an hrPKS, an ACS, and an ACDH) were identified clustered within the *P. fulvus* genome (Fig. 11).

**Fig. 12:**
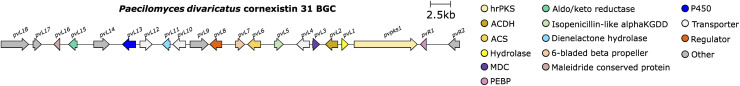
Cornexistin 31 BGC.

In addition, a gene (*bfL1*) encoding an enzyme with a putative hydrolytic function was identified, which is also homologous (35.48% identity) to the esterase from the asperlin BGC.^95^ Expression of the *P. fulvus* hrPKS, ACS and ACDH in *A. oryzae* did not produce any novel compounds, whereas these genes, with the addition of *bfL1*, produced the carboxylated anhydride 64 and its decomposition product, 57. This is contradictory to the results obtained by Oikawa and co-workers^90^ where the addition of a hydrolytic enzyme was not necessary for the production of monomers. Later work by Cox and co-workers^50^ investigating the cornexistin 31 pathway *via* gene deletion experiments, also suggested that the homologous hydrolase (*pvL1*) in the cornexistin BGC (Fig. 12) is essential, as no maleidride related compounds accumulated in the hydrolase deletion strain.

Interestingly, all confirmed and putative maleidride BGCs contain a hydrolase homologue, suggesting that it is important for the biosynthesis of maleidride compounds.^96^*In vitro* studies by Cox and co-workers^101^ showed that the *P. fulvus* hydrolase, *BfL1*, catalysed the hydrolysis of a series of a thiol esters, rather than being ACP-selective, therefore exactly how selectivity is controlled is unknown.^101^

**Scheme 18:**
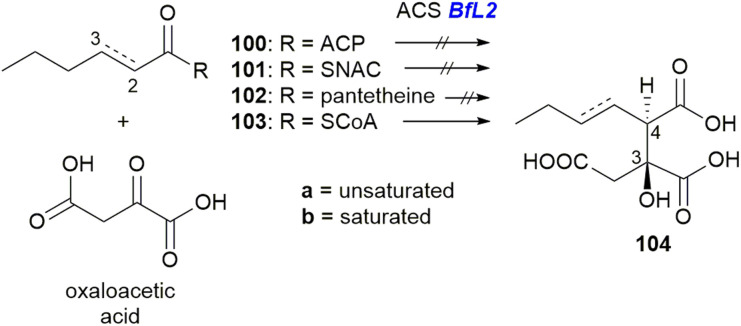
Turnover of substrate 103 and oxaloacetic acid to 104 by ACS *BfL2*.^101^

Investigations into the ACS and ACDH enzymes through *in vitro* characterisation have also been reported.^101^ Assays using both unsaturated (a) and saturated (b) versions of the substrates 100, 101, 102 and 103, with oxaloacetic acid and purified *BfL2* (ACS) showed that only the CoA thiol ester 103a/b could be turned over by *BfL2* (Scheme 18) to produce 104a/b.

Comparison of 104a to synthetic standards revealed that the enzyme product is exclusively the *anti* diastereomer.^101^

The synthesis of citrate is catalysed in most organisms by a *Si*-citrate synthase, with known *Re*-citrate synthases phylogenetically unrelated to *Si*-citrate synthases.^102^ A structural model of *BfL2* was built based on the primary metabolism citrate synthase from *Acetobacter aceti*,^103^ which is phylogenetically related to other *Si*-citrate synthases. Furthermore, the crystal structure of the *A. aceti* citrate synthase is bound to oxaloacetate and an acetyl CoA mimic in positions that should result in an *S* stereocentre.^101^ The structural model of *BfL2* showed that all of the residues involved in catalysis and binding oxaloacetate and acyl CoA are structurally highly conserved with the *A. aceti* citrate synthase.^101^ This led to the proposal that *BfL2* also creates a 3*S*-stereocentre, and thus ultimately an 3*S*,4*R* configuration.^101^ Cox and co-workers^101^ also suggested that differences in the configuration at the 4-position of 104 must be controlled by the geometry of the enoyl CoA intermediate.^101^ Recent *in silico* analysis of maleidride BGCs by Williams *et al.*^96^ has shown that many clusters contain an enoyl CoA isomerase, which may be involved in providing the appropriate substrate for the ACSs.

**Scheme 19:**
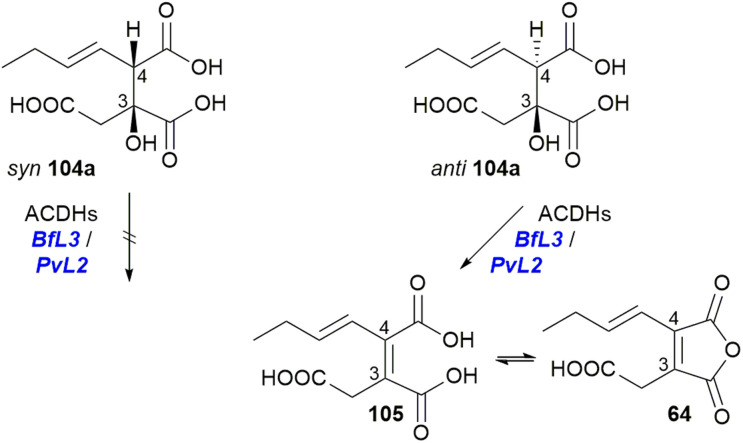
Turnover of substrate 104a by either *BfL3* or *PvL2* to the diacid 105 and anhydride 64.^101^

*In vitro* assays with purified ACDHs from the *P. fulvus* or *P. divaricatus* BGCs (*BfL3*/*PvL2*) demonstrated that only the *anti* diastereomer 104a can be dehydrated to produce the equilibrated products 105, the diacid, and 64, the anhydride (Scheme 19).^101^

### 5.2. Core genes for dimerisation

Comparison of the maleidride BGC from *P. fulvus* by Cox and co-workers^75^ to putative maleidride BGCs identified from genome sequences available on NCBI, as well as the putative phomoidride *e.g.*37 BGC^90^ revealed further genes in common. Each cluster encodes one or two proteins that have some similarity to ketosteroid isomerases (KSI-like) and one or two proteins that contain phosphatidylethanolamine-binding protein (PEBP) domains.^75^ Expression of the monomer forming genes (PKS, hydrolase, ACS and ACDH) with both KSI-like genes in the host *A. oryzae* led to the production of both byssochlamic acid 6 and agnestadride A 53 demonstrating that within the context of the *A. oryzae* genome, there are sufficient catalytic activities to perform both head-to-tail and head-to-side dimerisations of maleidride monomers, and that the KSI-like enzymes catalyse that dimerisation. The presence of both KSI-like enzymes appeared to be required for the dimerisation to occur *in vivo*. Addition of the two genes containing PEBP domains led to an over 20-fold increase in dimerised products.^75^

Further studies by Cox and co-workers^101^ showed that in contrast to the *in vivo* experiments, yeast cell-free extracts of either *P. fulvus* KSI-like enzyme are capable of catalysing dimerisation. Addition of the *P. fulvus* PEBP enzymes did not appear to appreciably increase yields of dimerised products, however the low-yielding nature of these experiments makes quantitative comparisons difficult.^101^ We have previously proposed that the KSI-like enzymes are renamed ‘maleidride dimerising cyclases’ (MDCs), as they alone are sufficient to perform the dimerisation reaction.^96^ All known and putative MDCs contain an NTF2 domain (nuclear transport factor 2 – IPR032710), which categorises them within the NTF2-like superfamily.^96^ This large group of proteins, which includes enzymes that have isomerase, cyclase, dehydratase and hydrolase activities, have low sequence identity but share a common structural fold that can be adapted to serve a range of functions.^104^

**Fig. 13:**
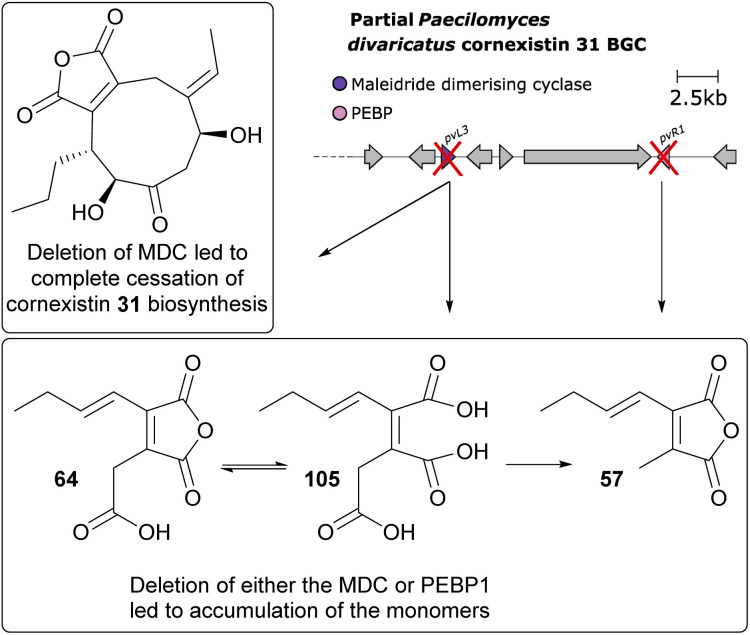
Overview of deletion of genes involved in dimerisation from the cornexistin 31 BGC.^50^

Further gene deletions to the cornexistin 31 producer, *P. divaricatus* corroborated these results, and suggested at least a supplementary role for the PEBP enzymes.^50^ Within the cornexistin BGC, only one MDC and one gene containing a PEBP domain are present (Fig. 12). Deletion of the MDC gene led to complete cessation of cornexistin 31 biosynthesis, with accumulation of the carboxylated anhydride monomer 64 and its spontaneous ring open form 105, which had not previously been detected from *P. divaricatus* extracts. Deletion of the gene containing the PEBP domain led to a decrease in the titre of cornexistin 31, and accumulation of 64, 105 and the decarboxylated monomer 57 (Fig. 13).^50^

**Fig. 14:**
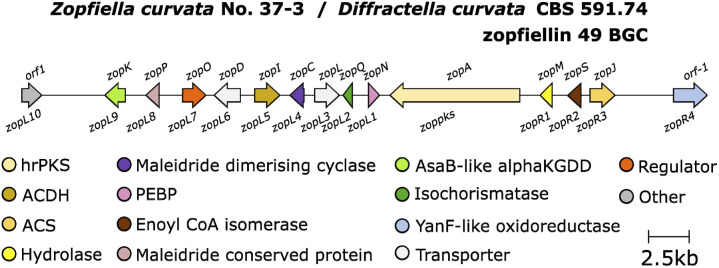
Zopfiellin 49 BGC.

Further research investigating the biosynthesis of zopfiellin 49 by Oikawa and co-workers^105^ identified a zopfiellin BGC (Fig. 14) from the genome of *Z. curvata*.

**Scheme 20:**
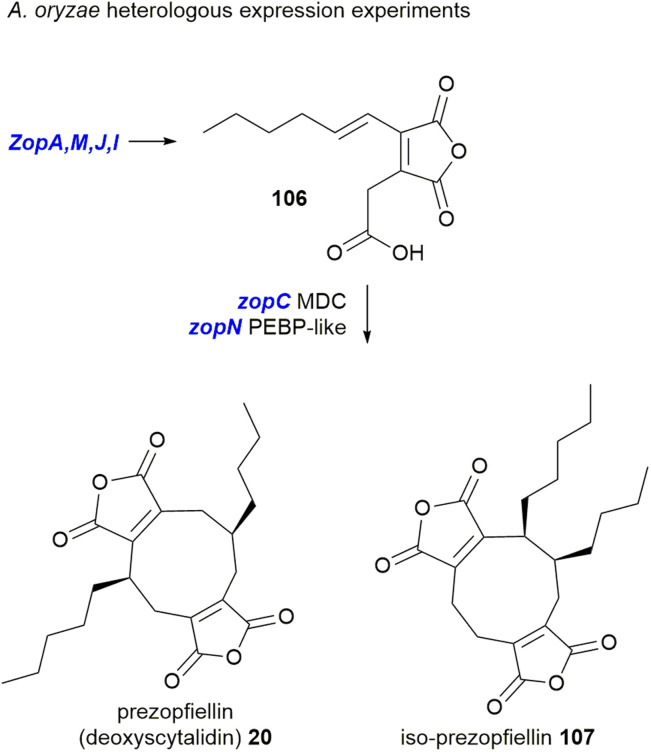
Heterologous expression of genes from the zopfiellin BGC led to the production of nonadrides.^105^

This work again demonstrated that the MDC and PEBP genes are involved in dimerisation of maleidride monomers; once introduced to an *A. oryzae* strain producing the zopfiellin monomer 106, two dimerised products were isolated, the nonadrides prezopfiellin 20 (which was identified as deoxyscytalidin 20 by Willis and co-workers^8^) and *iso*-prezopfiellin 107 (Scheme 20).^105^ It is notable that the mode of dimerisation for these nonadrides is different, *i.e.*: head-to-tail to produce deoxyscytalidin 20 and head-to-head (mode B) for iso-prezopfiellin 107 (see Scheme 17 for dimerisation types). This is the second known system where different modes of dimerisation can occur within the same pathway, the first being the biosynthesis of the nonadride byssochlamic acid 6 (head-to-tail dimerisation) and the heptadrides, agnestadrides A and B 53 and 54 (head-to-side dimerisation).^1^

No evolutionary relationship regarding mode of dimerisation appears to be displayed by the MDCs.^96^ The lack of close homologues to the MDCs constrains our ability to predict a mechanism for these enzymes, with crystallisation, modelling and mutation studies likely required to further our understanding of these unique enzymes. Until then, exactly how the MDCs control dimerisation, including apparently simultaneously catalysing different modes of dimerisation, remains cryptic.

The putative accessory role of the PEBP containing enzymes has been hypothesised to involve the chaperoning of unstable intermediates such as 1 and/or the known anionic binding ability of PEBP containing enzymes.^75,106^

### 5.3. Comparison of maleidride BGCs

**Fig. 15:**
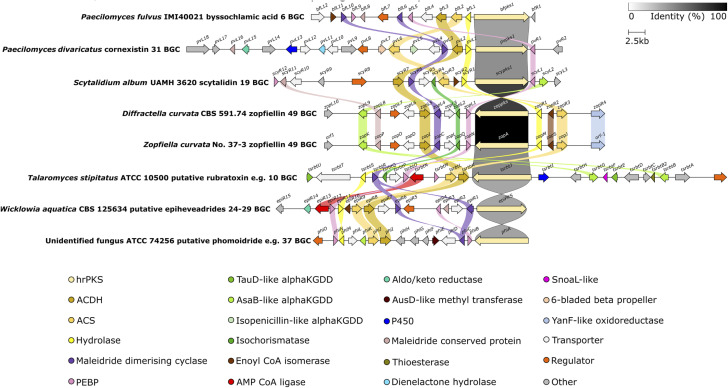
Clinker^107^ comparison between definitively linked maleidride BGCs (through gene knockout or heterologous expression), as well as those identified from the genomes of confirmed maleidride producing strains. The *T. stipitatus* cluster is included as it shares complete synteny with the *P. dangeardii* rubratoxin *e.g.*10 BGC, which is not publicly available.^96^ Links between homologous genes are shown using their specific colour, except for the PKSs where the links are shown according to the percentage identity (see identity scale bar). BGCs are aligned on the PKS and links between transport and regulatory genes have been removed for clarity. Figure reproduced from ref. 96.

To date there are six BGCs which have been linked to specific maleidrides through experimental approaches: the byssochlamic acid 6/agnestadrides *e.g.*53 BGC,^75^ the rubratoxins *e.g.*10 BGC,^37^ the cornexistin 31 BGC,^50^ two zopfiellin 49 BGCs,^8,105^ and the scytalidin 19 BGC.^8^ Two maleidride BGCs have been identified from confirmed maleidride producing strains – linked to phomoidrides *e.g.*37 (ref. 90) and epiheveadride 25 biosynthesis (Fig. 15).^96^ A further fourteen putative maleidride BGCs have been identified from publicly available genomes.^96^ Bioinformatic comparison of these maleidride BGCs supported the conserved core set of genes required for basic maleidride biosynthesis in all clusters – those encoding monomer biosynthesis – the hrPKS, the hydrolase, the alkylcitrate synthase and the alkylcitrate dehydratase, and those involved in dimerisation – the maleidride dimerising cyclases and the PEBP-like. In all cases, the clusters contain one or two MDC genes. Most clusters have one or two genes that contain a PEBP domain.^96^ The hypothesised ancillary nature of the PEBP enzymes does not preclude those clusters without genes that contain a PEBP domain from encoding maleidride biosynthesis.^96^

There are further sets of genes in common between the maleidride BGCs, some of which are common to many fungal natural product BGCs, the cytochrome P450s, α-ketoglutarate-dependent dioxygenases (αKGDDs), regulators and transporters, and some of which are more specific to maleidride BGCs, for example the isochorismatase-like, and a group of genes with sequence homology to each other, but with no characterised homologues (conserved maleidride proteins) (Fig. 15). Many of the genes which encode for catalytic enzymes are likely to be involved in post-dimerisation tailoring (see Section 5.4.2), however, the function of many others currently remains obscure.^96^

### 5.4. Genes responsible for maleidride structural diversification

#### 5.4.1. Monomer diversification

**Fig. 16:**
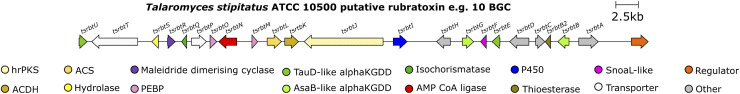
Putative rubratoxin *e.g.*10 BGC – the completely syntenous BGC from the *Talaromyces stipitatus* genome is shown, as the *P. dangeardii* sequence is not publicly available.

Amongst the maleidride PKSs linked to a specific maleidride compound, a tentative phylogenetic relationship between amino acid sequence and polyketide chain length has been shown, which may allow for chain length prediction in novel maleidride PKSs.^96^ Known maleidride monomers have variations only in chain length (triketide to hexaketide) and the degree of saturation in the polyketide chain. A potential exception are the rubratoxins, where a BGC has been identified from the genome of the rubratoxin *e.g.*10 producer *Penicillium dangeardii* (Fig. 16).^37^ Investigation of the rubratoxin pathway *via* gene deletions in *P. dangeardii* and *in vitro* studies suggested that one of the monomers for rubratoxin biosynthesis is ω-hydroxylated prior to dimerisation.^37^

**Scheme 21:**
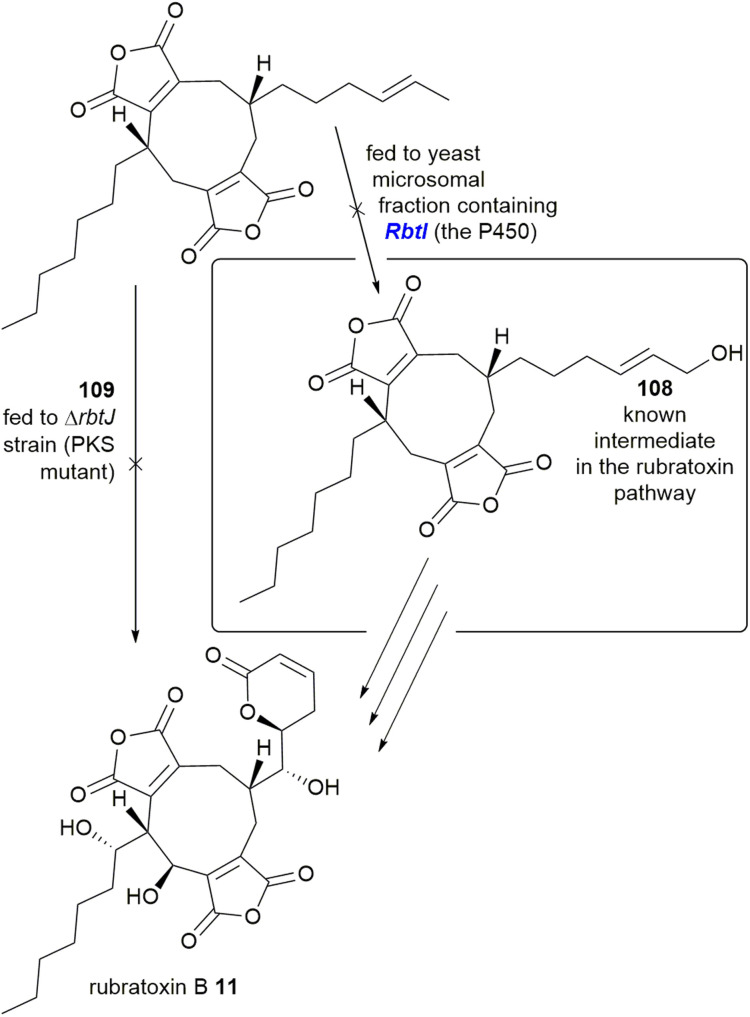
Experiments to attempt to determine the function of *RbtI*, a P450 from the rubratoxin BGC.^37^

Deletion of a P450 within the rubratoxin BGC, *rbtI*, produced a range of dimeric nonadrides without the terminal hydroxyl group identified in the known intermediate 108. The deoxy analogue 109 of 108 was proposed to be the substrate for *RbtI*, however feeding of 109 to the PKS deletion strain did not restore rubratoxin A 10 or B 11 biosynthesis. Additionally, no hydroxylation was detected upon feeding of 109 to cell free extract of a yeast strain expressing *RbtI* (Scheme 21). The Hu, Yu and Tang groups^37^ proposed that the true substrate of *RbtI* is one of the monomers, however direct evidence for this was not provided.^37^ Phylogenetic analysis of an orthologue, TsRbtI, from *T*. *stipitatus*, demonstrated that this enzyme clades with other P450s which possess a similar function, providing further evidence that this enzyme catalyses ω-hydroxylation.^96^

#### 5.4.2. Post-dimerisation diversification

##### 5.4.2.1. Cytochrome P450s

Cytochrome P450s are oxidative enzymes that are common in fungal natural product BGCs, interestingly very few maleidride clusters contain a P450. One is *RbtI*, discussed in Section 5.4.1, which appears to be involved in pre-dimerisation diversification.^37^

**Scheme 22:**
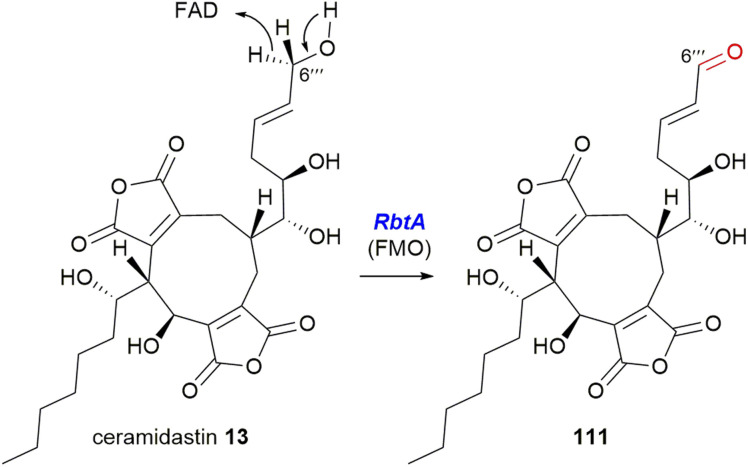
Proposed route for the production of cornexistin from dihydrocornexistin according to Cox and co-workers *via* the cytochrome P450 *PvL13*.^50^

*PvL13* is a P450 encoded within the cornexistin 31 BGC (Fig. 12). Work by Cox and colleagues^50^ to investigate the biosynthetic pathway to the herbicidal compound cornexistin 31, produced a mutant strain with a deletion of the P450, Δ*pvL13*. This strain accumulated the compound dihydrocornexistin 34, and neither the hemiacetal 110 nor cornexistin 31 were detected. This led Cox and co-workers to propose that the C-6 double bond is introduced *via* a hydroxylation at C-6, though only the more stable hemiacetal 110 was isolated. The exact mechanism for conversion of 110 to cornexistin 31 is unclear, but the P450 may be multifunctional (Scheme 22).^50^

##### 5.4.2.2. Flavin-dependent monooxygenase

**Scheme 23:**
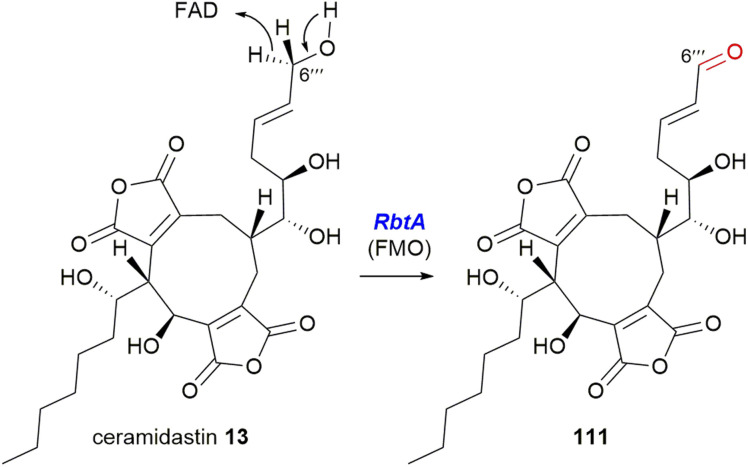
Oxidation of ceramidastin 13 by *RbtA*, a flavin-dependent monooxygenase.^37^

The Hu, Yu and Tang groups^37^ investigating the rubratoxin biosynthetic pathway had isolated a shunt compound with an α,β-unsaturated aldehyde at C-6′′′, which suggested that the production of the carboxylate required for the mature lactone moiety in rubratoxins A 10 and B 11, might proceed stepwise *via* an aldehyde. The rubratoxin BGC is the only known or putative maleidride BGC to contain a flavin-dependent monooxygenase (FMO), *RbtA* (Fig. 16).^96^ Bioinformatic analysis of this enzyme shows that it contains a berberine-bridge enzyme (BBE) domain (IPR012951) and an PCMH-type (*p*-cresol methylhydroxylase) FAD-binding (flavin adenine dinucleotide) domain (IPR016166). A mutant strain, Δ*rbtA*, was no longer able to produce rubratoxins A 10 or B 11, but accumulated the known compound ceramidastin 13, suggesting *RbtA* is involved in the oxidation of the C-6′′′ alcohol to the aldehyde. *RbtA* was expressed and purified from *Saccharomyces cerevisiae* and subjected to assays with ceramidastin 13 as a substrate and FAD which led to the production of 111, confirming the role of *RbtA* in the rubratoxin biosynthetic pathway (Scheme 23).^37^

##### 5.4.2.3. Ferric reductase

Within the rubratoxin BGC is a gene encoding a ferric reductase, *RbtH* (Fig. 16), consisting of three domains – a ferric reductase like transmembrane domain (IPR013130), a ferredoxin-like (FR) domain, a ferredoxin reductase (FNR) like domain as well as binding sites for [Fe_2_S_2_], FAD and NADH (reduced nicotinamide adenine dinucleotide). The Hu, Yu and Tang groups^37^ produced a mutant Δ*rbtH* strain which accumulated rubratoxin B 11, with the cessation of rubratoxin A 10 biosynthesis, suggesting that *RbtH* selectively reduces the C-8′ carbonyl to a corresponding hydroxyl group. Additionally whole cell bioconversion assays using *RbtH* expressed in *S. cerevisiae*, subjected to rubratoxin B 11, showed complete conversion to rubratoxin A 10.^37^

Although other maleidrides contain the γ-hydroxybutenolide motif present in rubratoxin A 10 (for example phomoidrides A 36 and C 39, tetrahydroepiheveadride 27, dihydroepiheveadride 24 and dihydrobyssochlamic acid 9), no homologous ferric reductase is present in any other confirmed or putative maleidride BGC.^96^ Furthermore, this reduction is not seen in the structurally related rubratoxin C 12 and ceramidastin 13, which might suggest the BGCs encoding the biosynthesis of 12 and 13 do not contain *rbtH* homologues.

##### 5.4.2.4. α-Ketoglutarate-dependent dioxygenases

Many maleidride BGCs contain α-ketoglutarate-dependent dioxygenases (αKGDDs). These are versatile enzymes that catalyse various C–H bond activation reactions, including hydroxylation, desaturation, ring expansion/contraction, dealkylation, epoxidation, epimerisation, halogenation, cyclisation and peroxide formation.^108^ Even within the maleidride clusters, characterised αKGDDs catalyse hydroxylation (*PvL5*,^50^*ScyL2*,^8^*RbtB*, *RbtG*, *RbtE*, and *RbtU*^37^), and oxidative ring contraction (*ZopK*^105^/*ZopL9*^8^). αKGDDs lack sequence identity, but possess structural similarities, including a core double-stranded β-helix fold that binds Fe and the co-substrate αKG *via* a conserved HXD/E⋯H motif.^109^ The confirmed maleidride αKGDDs fall into three distinct groups, those in the taurine dioxygenase TauD-like superfamily (IPR042098), the isopenicillin N synthase-like (IPR027443), and the AsaB-like (IPR044053).^96^

**Scheme 24:**
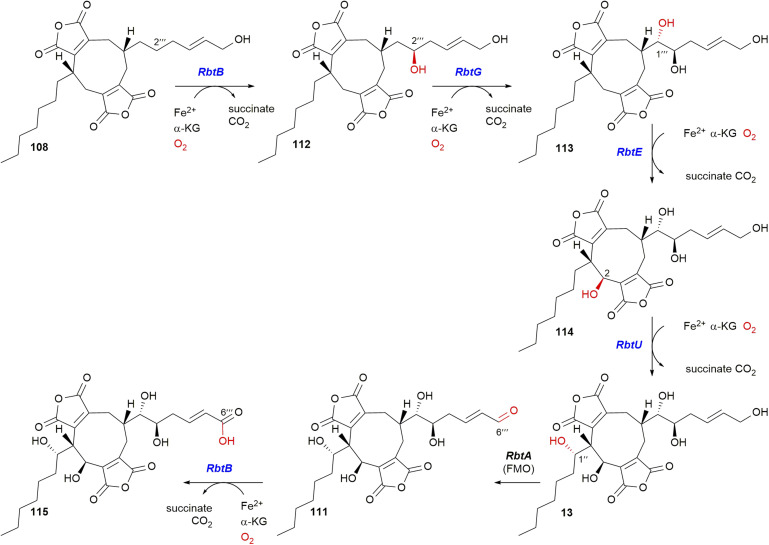
Summary of the reactions catalysed by αKGDD enzymes within the rubratoxin pathway based on experiments by the Hu, Yu and Tang groups.^37^

The rubratoxin BGC contains four αKGDDs, two TauD-like, *RbtE* and *RbtU*, and two from the AsaB-like IPR044053 group, *RbtB* and *RbtG* (Fig. 16). The activities of these enzymes were deduced *via* gene knockout, chemical complementation and *in vitro* enzyme assays (Scheme 24).^37^ An *in vitro* experiment using *E. coli* expressed and purified *RbtB* demonstrated that the presence of αKG and Fe^2+^ is a requirement for catalysis. Further assays for *RbtG*, *RbtE* and *RbtU* assumed the necessity of αKG and Fe^2+^. Interestingly *RbtB* was shown to be bifunctional and catalyse both C-2′′′ hydroxylation to give 112, and the C-6′′′ oxidation of 111 to give 115 (Scheme 24).^37^

**Scheme 25:**
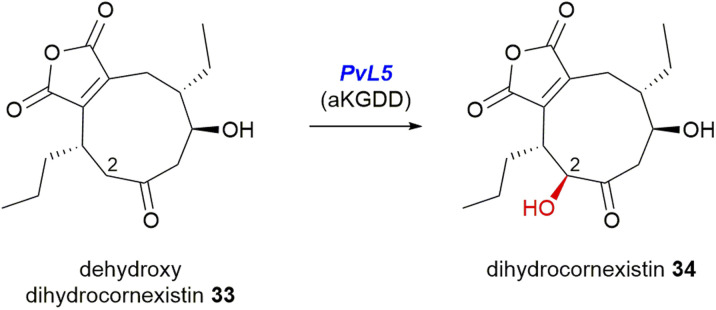
The *pvL5* mutant strain accumulated dehydroxydihydrocornexistin 33, suggesting *PvL5* is involved in C-2 ring hydroxylation, according to experiments by Cox and co-workers.^50^

*PvL5* of the cornexistin 31 pathway is the only αKGDD enzyme from the maleidride BGCs which is isopenicillin N synthase-like (IPR027443).^96^ A gene knockout of *pvL5* (Fig. 12) accumulated dehydroxydihydrocornexistin 33, suggesting that the *PvL5* enzyme is involved in ring hydroxylation at C-2 (Scheme 25).^50^

**Scheme 26:**
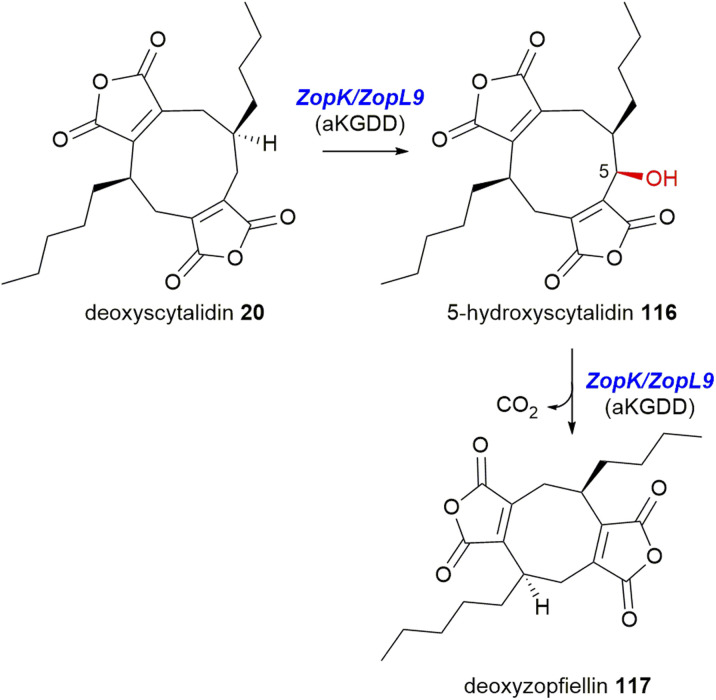
Proposed stepwise catalysis of the ring contraction required for zopfiellin 49 biosynthesis.^8,105^

In 2020, both Oikawa and co-workers^105^ and Willis and co-workers^8^ demonstrated that for the zopfiellin 49 biosynthetic pathway, αKGDD enzymes (the orthologues *ZopK*/*ZopL9* – within the AsaB-like IPR044053 group) are responsible for the oxidative ring contraction required for the formation of the octadride, zopfiellin 49, *via* successive oxidation of the nonadride 20, to 116, followed by a final conversion to the octadride deoxyzopfiellin 117, albeit at low titre (Scheme 26).^8,105^

Both groups identified putative maleidride BGCs from the genomes of *Z*. *curvata* No. 37-3,^105^ and from *D*. *curvata* CBS 591.74 respectively.^8^ Oikawa and co-workers^105^ undertook heterologous production experiments using the heterologous host, *A. oryzae*. Expression of all the genes predicted to produce a simple nonadride led to the accumulation of 20 (see Scheme 20). Addition of the αKGDD enzyme *ZopK* to this strain led to two new products by LCMS analysis. The major product was shown to be the nonadride, 116, whilst small amounts of the octadride, deoxyzopfiellin 117 were also detected.

To characterise the activity of the αKGDD enzyme further, both Oikawa and co-workers^105^ and Willis and co-workers^8^ performed *in vitro* assays with the *ZopK*/*ZopL9* enzymes using αKG, Fe^2+^ and substrate. Willis and co-workers^8^ had determined through gene disruption and chemical complementation experiments that the substrate for *ZopL9* is in fact deoxyscytalidin 20, a known nonadride isolated from *Scytalidium* sp.^40^ Both groups showed that 20 was turned over by *ZopK*/*ZopL9* to produce 116 and trace amounts of deoxyzopfiellin 117.^8,105^ Assays using *ZopK*/*ZopL9* with the substrate 116 led to increased turnover (albeit still low titre) to deoxyzopfiellin 117. This confirms the stepwise catalysis by the αKGDD enzymes *ZopK*/*ZopL9* to produce the octadride deoxyzopfiellin 117 from the nonadride 20*via* an oxidative ring contraction (Scheme 26).^8,105^ However the low titre of the ring contraction product, deoxyzopfiellin 117, demonstrated in both the *in vivo* heterologous expression experiments,^105^ and the *in vitro* assays^8,105^ suggests that perhaps another enzyme(s) might be required to support this activity.

Bioinformatic analysis by Willis and co-workers^8^ showed that the closest characterised homologue of *ZopL9* is the gibberellin desaturase DES (S0E2Y4.1). This enzyme catalyses the desaturation of gibberellin A4 to gibberellin A7, although it can also perform hydroxylations.^110^ Interpro analysis shows that *ZopK*/*L9* and DES share a currently unnamed domain: PTHR34598:SF3.

**Fig. 17:**
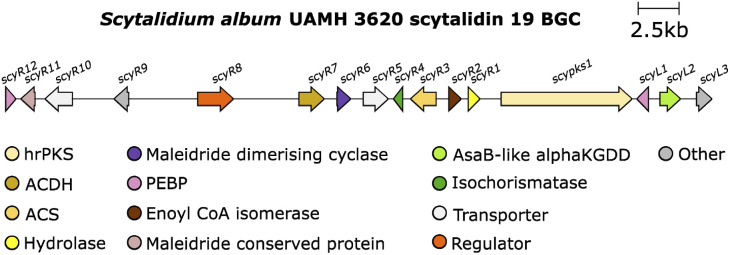
Scytalidin 19 BGC.

The study by Willis and co-workers^8^ also investigated an αKGDD enzyme from the scytalidin 19 pathway. The authors identified a putative maleidride BGC from the genome of the scytalidin producer, *S. album* UAMH 3620 (Fig. 17).

**Scheme 27:**
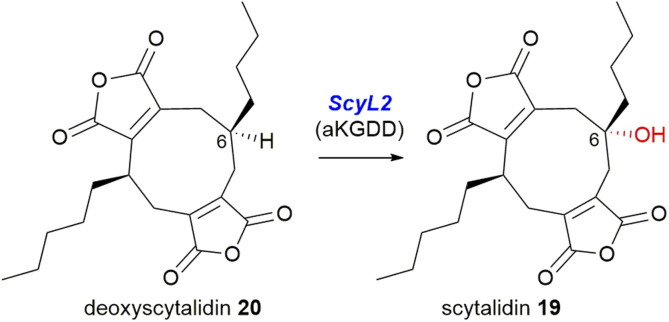
The *scyL2* mutant strain accumulated deoxyscytalidin 20, which suggests the αKGDD enzyme *ScyL2* performs the 6-hydroxylation required to produce scytalidin 19.^8^

The direct comparison of the BGCs for scytalidin 19 and zopfiellin 49 revealed that each cluster encodes an αKGDD enzyme, the aforementioned *ZopL9*, and *ScyL2*, which although both fall within the AsaB-like IPR044053 group, have low sequence identity, suggesting differing function (∼25% identity).^8^ Mutant strains of *S. album* were generated with a deletion of the *scyL2* gene, which accumulated deoxyscytalidin 20, suggesting that *ScyL2* is responsible for the hydroxylation at C-6 (Scheme 27).^8^

**Scheme 28:**
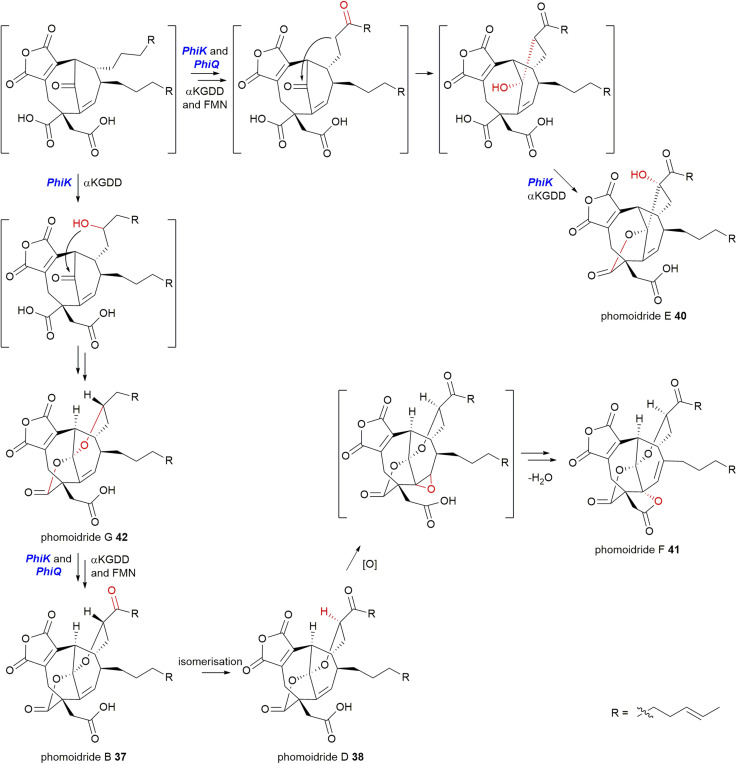
Proposed pathway to the phomoidrides B 37, D 38, E 40, F 41, and G 42 according to Hu and co-workers.^53^ Intermediates in square brackets are predicted and have not been isolated.

Willis and co-workers^8^ also identified that *PhiK*, an uncharacterised protein encoded within the phomoidride BGC, is homologous to *ScyL2*, *RbtG* and *ZopK*/*L9*. It is likely that this enzyme catalyses one or more of the post-dimerisation oxidative steps required to produce the mature phomoidride structure.^8^ The recent discovery of phomoidrides E 40, F 41, and G 42 prompted Hu and co-workers^53^ to propose that *PhiK* undertakes multiple oxidations in concert with *PhiQ*, an FMN binding oxidoreductase, to synthesise phomoidrides B 37, D 38, E 40, F 41 and G 42 (Scheme 28), however no molecular evidence has been provided.

## 6. Overview of maleidride compounds

**Fig. 18:**
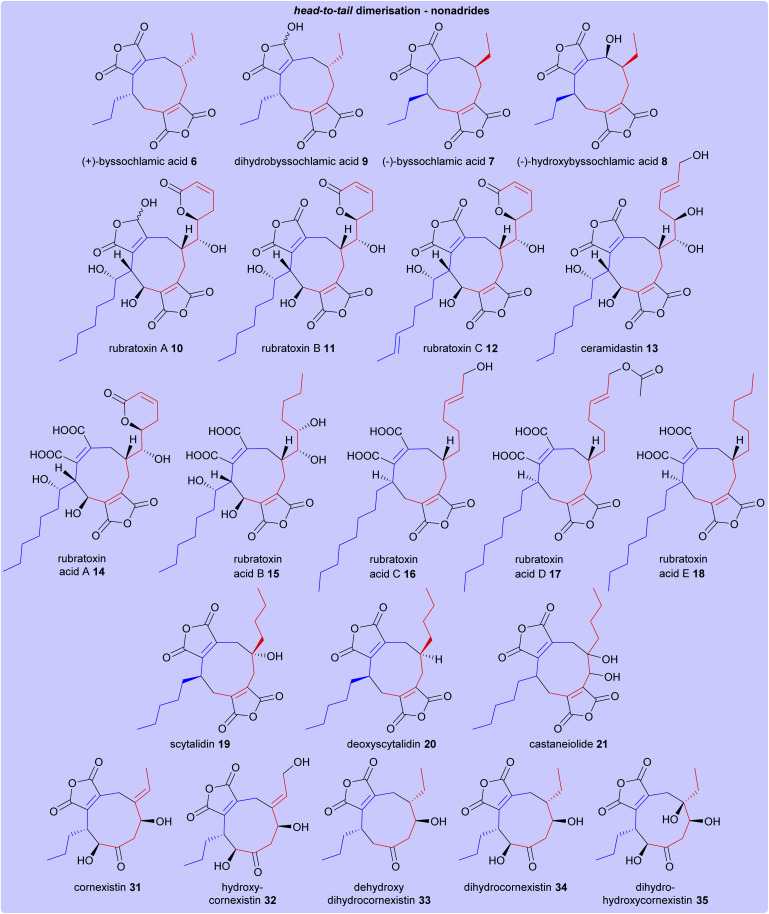
Structures of head-to-tail dimerised nonadrides. Heterodimerisations of monomers A 1 and C 3 are depicted according to the colours shown in Scheme 17.

**Fig. 19:**
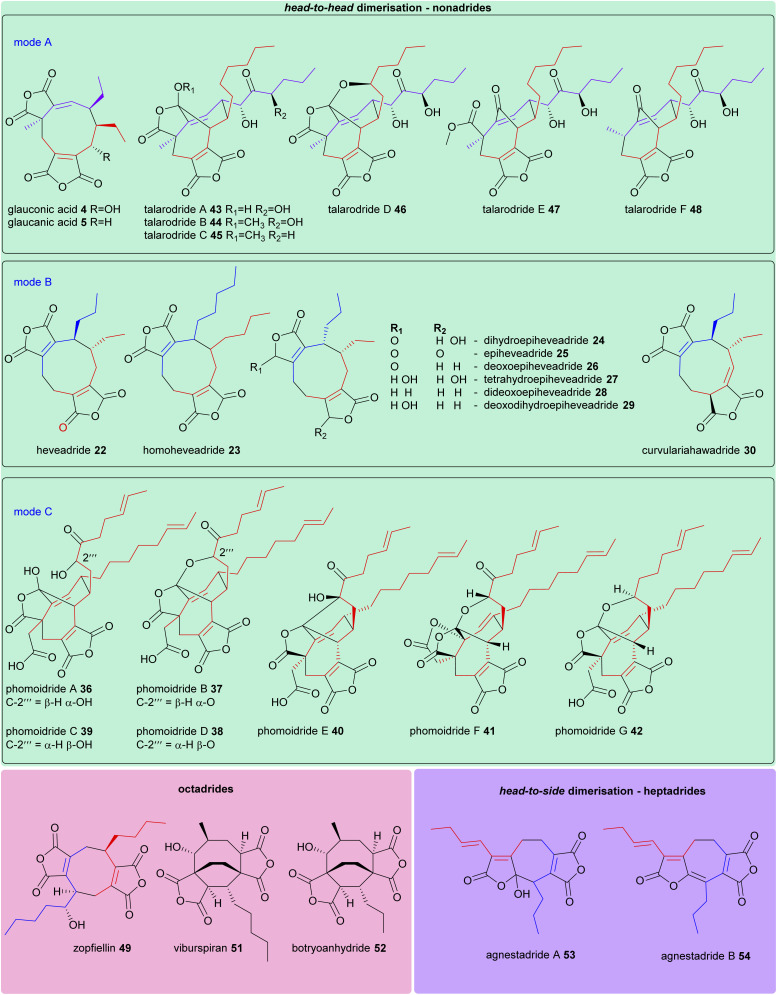
Structures of head-to-head dimerised nonadrides, as well as octadrides and heptadrides. Where mode of dimerisation can be deduced, and therefore which monomers have dimerised (homo- and hetero-dimerisations of monomers A 1, B 2, and C 3), these are depicted according to the colours shown in Scheme 17.

The structures of all maleidride compounds discussed in this review have been classified in Fig. 18 and 19 according to their mode of dimerisation, to demonstrate the structural relationships between these compounds. Furthermore, their known or predicted monomer chain length, producing species, and any known bioactivities have been collated in Table 1.

**Table 1:** Maleidrides classified according to the size of central ring structure and mode of dimerisation. The producing fungus, the predicted size of the monomer unit, and known bioactivities are shown

Maleidride type	Dimerisation	Mode	Compound	Fungus	Monomer	Bioactivity
Nonadride	Head-to-head	A	Glauconic acid 4	Various *Talaromyces* species^100^	Triketide	Unknown
			Glaucanic acid 5		Triketide	Unknown
			Talarodride A 43	*Talaromyces* sp. HDN1820200 (ref. 57)	Pentaketide	Antibacterial^57^
			Talarodride B 44		Pentaketide	Antibacterial^57^
			Talarodride C 45		Pentaketide	Unknown
			Talarodride D 46		Pentaketide	Unknown
			Talarodride E 47		Pentaketide	Unknown
			Talarodride F 48		Pentaketide	Unknown
Nonadride	Head-to-head	B	Heveadride 22	*Bipolaris heveae* CBS 241.92 (ref. 42)	Triketide	Antifungal^44^
			Homoheveadride 23	*Cladonia polycarpoides* nyl. in Zwackh^43^	Tetraketide	Unknown
			Dihydroepiheveadride 24	*Wicklowia aquatica* CBS 125634 (ref. 45)	Triketide	Antifungal^44^
			Epiheveadride 25		Triketide	Antifungal^44^
			Deoxoepiheveadride 26		Triketide	Antifungal^45^
			Tetrahydroepiheveadride 27		Triketide	Unknown
			Dideoxoepiheveadride 28		Triketide	Unknown
			Deoxodihydroepiheveadride 29		Triketide	Unknown
			Curvulariahawadride 30	*Curvularia* sp. MFLCC12-0192 (ref. 46)	Triketide	Nitric oxide production inhibitory activity^46^
Nonadride	Head-to-head	C	Phomoidride A 36	Unidentified fungus ATCC 74256 (ref. 51–53)	Hexaketide	Squalene synthase and ras farnesyl transferase inhibitory activities^51^
			Phomoidride B 37		Hexaketide	Squalene synthase and ras farnesyl transferase inhibitory activities^51^
			Phomoidride C 39		Hexaketide	Unknown
			Phomoidride D 38		Hexaketide	Unknown
			Phomoidride E 40		Hexaketide	Cytotoxic against HeLa and p388 cells^53^
			Phomoidride F 41		Hexaketide	Unknown
			Phomoidride G 42		Hexaketide	Unknown
Nonadride	Head-to-tail		(+)-Byssochlamic acid 6	Various *Paecilomyces* species^48^	Triketide	Unknown
			Dihydrobyssochlamic acid 9	*Paecilomyces fulvus* IMI40021 (ref. 1)	Triketide	Unknown
			(−)-Byssochlamic acid 7	*Phomopsis* sp. K38 (ref. 18 and 19)	Triketide	Unknown
			(−)-Hydroxybyssochlamic acid 8		Triketide	Cytotoxic against HEp-2 and HepG2 cells^19^
			Rubratoxin A 10	Various *Talaromyces* species^100^	Pentaketide	PP2A inhibitor^26^
			Rubratoxin B 11		Pentaketide	Antitumour activity^30^
			Rubratoxin C 12		Pentaketide	Weak activity against human cancer cell lines^25^
			Ceramidastin 13	*Penicillium* sp. Mer-f17067 (ref. 31)	Pentaketide	Ceramidase inhibitor^31^
			Rubratoxin acid A 14	*Talaromyces purpurogenus* ^34^	Pentaketide	Nitric oxide production inhibitory activity^34^
			Rubratoxin acid B 15		Pentaketide	Unknown
			Rubratoxin acid C 16		Pentaketide	Unknown
			Rubratoxin acid D 17		Pentaketide	Unknown
			Rubratoxin acid E 18		Pentaketide	Unknown
			Scytalidin 19	*Scytalidium album* UAMH 3620 and UAMH 3611 (ref. 40)	Tetraketide	Antifungal^39^
			Deoxyscytalidin 20		Tetraketide	Unknown
			Castaneiolide 21	*Macrophoma castaneicola* M1-48 (ref. 41)	Tetraketide	Wilting in chestnut leaves^41^
			Cornexistin 31	*Paecilomyces divaricatus* ^47,49,50^	Triketide	Herbicidal^35,49^
			Hydroxycornexistin 32		Triketide	Herbicidal^35,49^
			Dehydroxydihydrocornexistin 33		Triketide	Unknown
			Dihydrocornexistin 34		Triketide	Unknown
			Dihydrohydroxycornexistin 35		Triketide	Unknown
Octadride	Head-to-tail		Zopfiellin 49	*Zopfiellia curvata* no. 37-3 (ref. 70) and *Diffractella curvata* CBS 591.74 (ref. 8), *Zopfiella curvata* no. 37-3 (ref. 70)	Tetraketide	Antifungal^36,70,71^
	Unknown		Viburspiran 51	*Cryptosporiopsis* sp. 8999 (ref. 72)	Unknown	Antifungal^72^
	Unknown		Botryoanhydride 52	Unidentified fungus BCC 54265 (ref. 73)	Unknown	Weak cytotoxicity to cancer cell-lines^73^

Heptadride	Head-to-side		Agnestadride A 53	*Paecilomyces fulvus* IMI40021 (ref. 1)	Triketide	Unknown
	Head-to-side		Agnestadride B 54		Triketide	Unknown

## 7. Conclusions

Since the first maleidride isolation in the 1930s,^9^ exactly how these compounds are formed have posed a challenge to our biosynthetic understanding, with increasing insight leading to the potential to synthesise and manipulate their structures in a rational manner. The core ring of 7-, 8- or 9-carbons is unusual in nature, and this class of compound has received growing interest as more representatives have been isolated, particularly given that the majority have important biological activities.^2^

Recent genetic and biochemical studies^50,75,90,101,105^ have added support to the original feeding studies^56,76–78^ showing that the monomer for the maleidrides is derived from an oxaloacetate cross-linked *via* its β carbon to the β carbon of a polyketide. The core set of enzymes responsible for formation of the monomer have been characterised: a highly reducing-PKS, a hydrolase, an alkylcitrate synthase and an alkylcitrate dehydratase.^75,90,96,101^ Moving beyond the monomer, the core enzyme required for dimerisation, and therefore ultimately controlling the structure of the mature maleidride, is the maleidride dimerising cyclase.^50,75,96,101,105^ This coupling reaction appears to be aided by the PEBP-like enzymes, although their exact role is currently obscure.^50,75,96,101,105^ The precise detail of how cyclisation is controlled remains cryptic, at present it is not possible to predict whether a biosynthetic gene cluster will deliver dimers showing head-to-head, head-to-tail or head-to-side modes of cyclisation, highlighting that there is still much to be discovered in this type of pathway.

In terms of the octadrides, we now have a far better understanding of how the octadride zopfiellin 49 is formed *via* a ring-contraction, with the oxidative elimination of a ring-carbon by an α-ketoglutarate dependent dioxygenase, converting the nonadride precursor to the octadride.^8,105^ It is yet to be determined whether the *ZopK*/*ZopL9* enzyme responsible for this step of zopfiellin 49 biosynthesis can be modified to ring-contract other nonadrides. Furthermore, with only limited yields recovered from both *in vitro* and *in vivo* reactions, a question remains as to whether additional, as yet unidentified, enzymes are required to elevate the yield of this type of reaction.^8,105^

Various modes of post-cyclisation tailoring have been highlighted and, given the ongoing discovery of new maleidride BGCs from sequence data hinting at unidentified members of this class,^96^ we expect the range of modifications available to continue to increase. The maleidrides are a challenging, but rewarding class of fungal natural product and the increasing knowledge about their biosynthesis raises interesting possibilities for combining synthetic biology approaches with semi-synthetic chemistry to deliver a wide range of maleidrides for future pharmacological assessment.

## 8. Author contributions

KW drafted the majority of the manuscript, with help from AJS. KMJdMS, AMB, RJC and CLW edited the manuscript with AMB, RJC and CLW contributing short sections.

## 9. Conflicts of interest

RJC is an Editor for the Special Issue “Engineering Fungal Biosynthetic Pathways”.

## Supplementary Material
